# An Unsupervised Classifier for Whole-Genome Phylogenies, the Maxwell© Tool

**DOI:** 10.3390/ijms242216278

**Published:** 2023-11-13

**Authors:** Joël Gardes, Christophe Maldivi, Denis Boisset, Timothée Aubourg, Jacques Demongeot

**Affiliations:** 1 Orange Labs, 38229 Meylan, France; joel.gardes@orange.com (J.G.); christophe.maldivi@orange.com (C.M.); denis.boisset@orange.com (D.B.); 2 Faculty of Medicine, Université Grenoble Alpes, AGEIS EA 7407 Tools for e-Gnosis Medical, 38700 La Tronche, France; timotheeaubourg@gmail.com

**Keywords:** unsupervised classifier, maxwell classifier, Burrows–Wheeler compression transform, normalized compression distance (NCD), Vitányi distance, phylogenetic trees

## Abstract

The development of phylogenetic trees based on RNA or DNA sequences generally requires a precise and limited choice of important RNAs, e.g., messenger RNAs of essential proteins or ribosomal RNAs (like 16S), but rarely complete genomes, making it possible to explain evolution and speciation. In this article, we propose revisiting a classic phylogeny of archaea from only the information on the succession of nucleotides of their entire genome. For this purpose, we use a new tool, the unsupervised classifier Maxwell, whose principle lies in the Burrows–Wheeler compression transform, and we show its efficiency in clustering whole archaeal genomes.

## 1. Introduction

There are numerous algorithms of classification, supervised or otherwise. In the set of supervised algorithms, we can find several types of classifiers, like k-means (k-nearest neighbors [1,2,3]) and SVM (support vector machine [4,5]) methods, and examples include neural networks with the Hopfield [6,7,8] or Boltzmann [9] approach and deep learning [10] for the unsupervised type (see also Supplementary Material Table S1 for other classifiers). The Maxwell Algorithm of Clustering (MAC) belongs to the last category. 

This MAC classification tool uses a totally reversible compression method (which is particularly suitable for the health sector), and applications have already been undertaken in genetics, for problems of storage or sequence recognition [11]. Here, the challenge is different: it consists of classifying nucleotide sequences of complete genomes, of distinct size and species, without any prior indication of co-evolution, to see if it is possible to obtain clusters in agreement with the existing phylogenies, which are, in general, based on specific RNAs (such as 16S ribosomal RNA) or proteins important for the biosynthesis or degradation of RNAs (such as RNase P1).

In Section 2, we will present the methodology related to the Burrows–Wheeler transform and Vitányi distance used in different MAC steps. Then, in Section 3, we describe some genomic applications, and in Section 4 and Section 5, we discuss these results and we conclude by opening some perspectives for future works.

## 2. Biological Context

### 2.1. Gene Translation for Building Proteins

**Figure 1:**
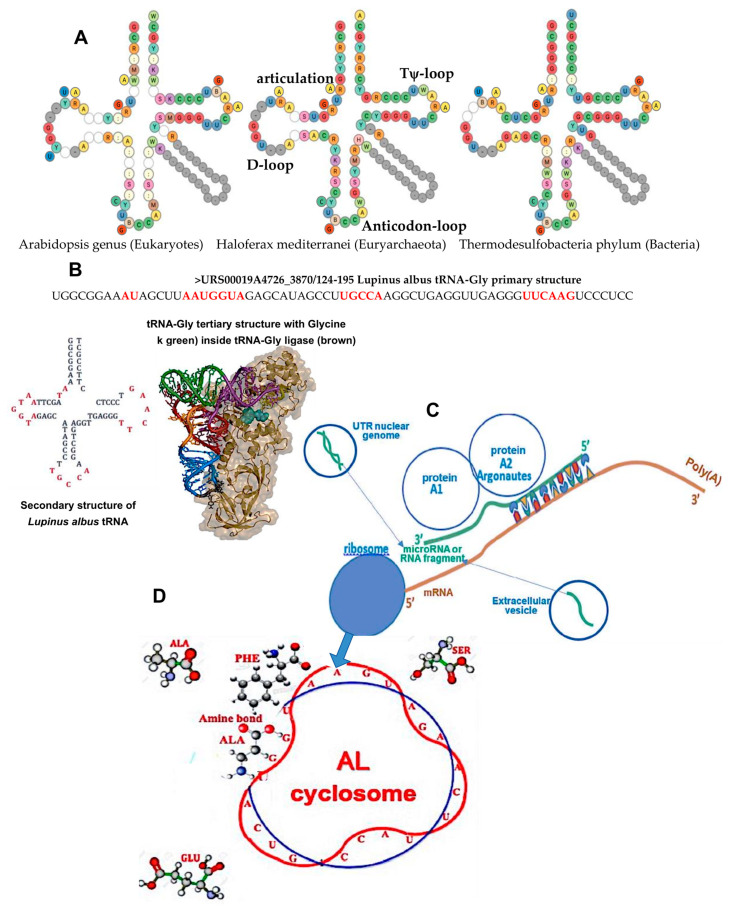
(**A**) tRNA-Gly^GCC^ secondary structure of eukaryotes (Arabidopsis), archaea (Haloferax) and bacteria (Thermodesulfobacterium) [12]. (**B**) The primary, secondary and tertiary structures of the tARN-Gly^GCC^ of *Lupinus albus* with its D-loop, Anti-codon loop, Ty-loop and articulation (in red). (**C**) Translation of an mRNA in the ribosome compared to (**D**) building of the peptidic dimer Phe-Ala catalyzed by the archetypal loop AL.

The tRNA-Gly^GCC^ secondary structures in different domains (eukaryotes, archaea and bacteria) contain invariant loops: D-loop, Anti-codon loop, Tψ-loop and articulation, whose sequence called AL (archetypal loop) is AAUGGUACUTGCCAUUCAAGAUG. AL is compatible with the loop sequence of tRNA-Gly^GCC^ of *Lupinus albus* (Figure 1). 

### 2.2. Combinatorial Properties of AL

**Figure 2:**
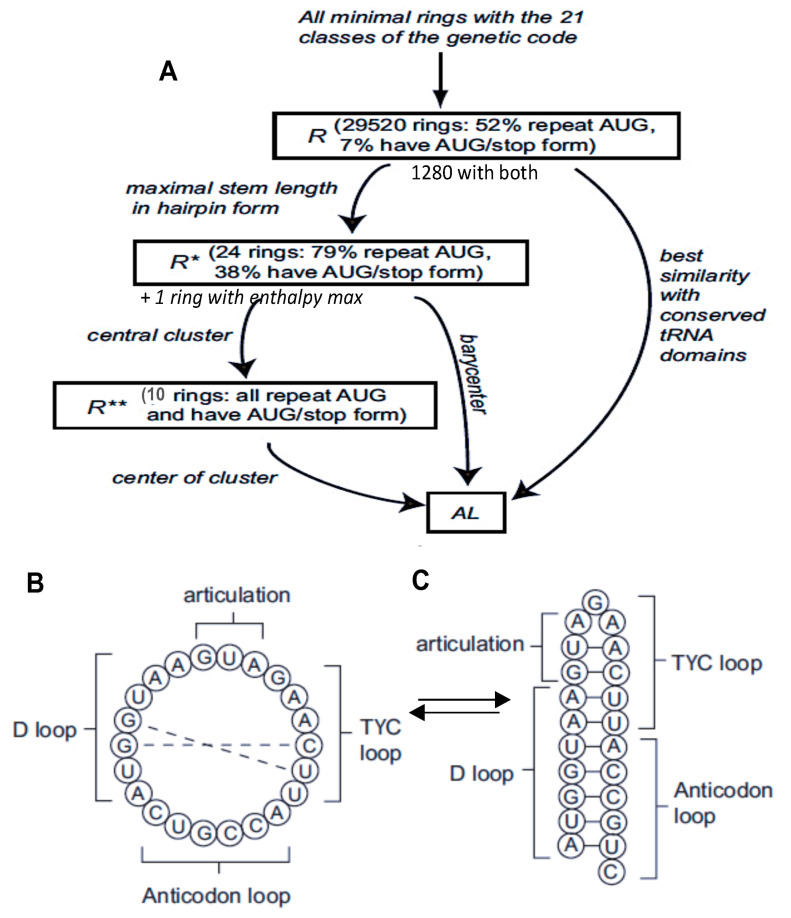
(**A**) Schematic summary of the search for the sequence AL, where rule R is “twice AUG”, rule R* is “existence of a hairpin shape of maximum enthalpy (requiring a complementarity of two halves of the loop” and R** is “repeat AUG and have AUG-stop form”; (**B**) circular shape of AL; (**C**) hairpin shape of AL.

**Figure 3:**
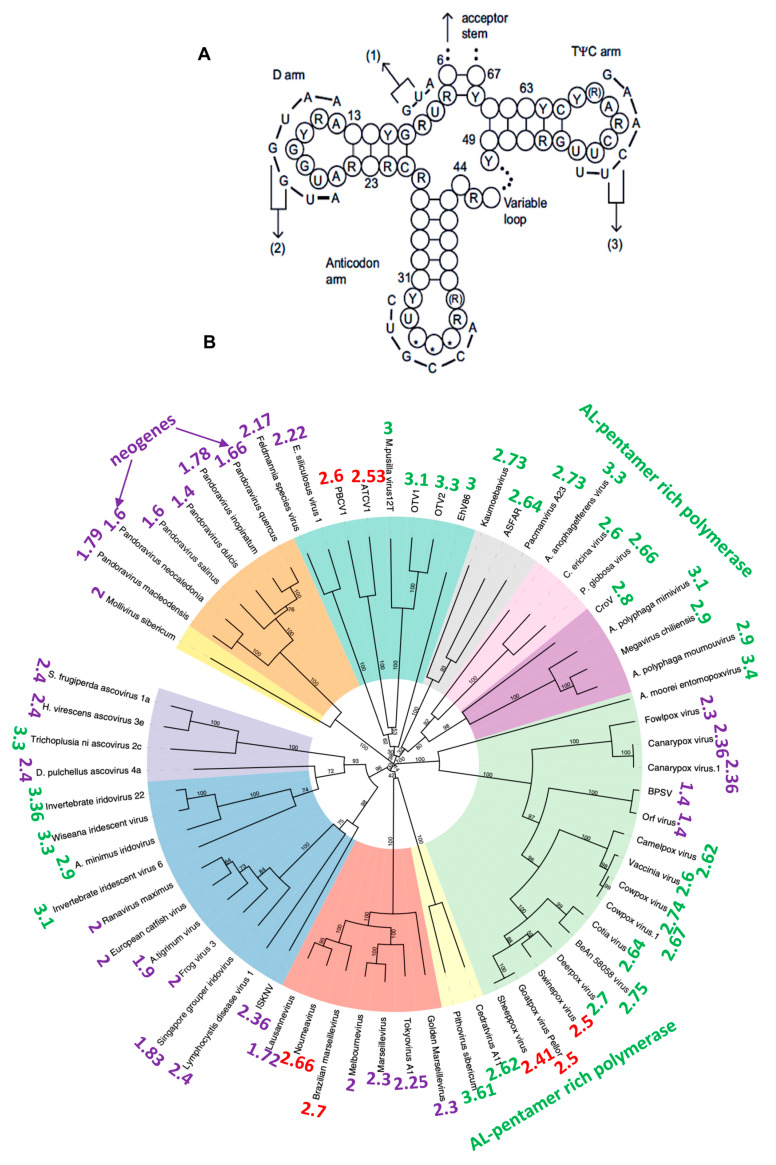
(**A**) tRNA general architecture with AL matching on the loops; (**B**) giant virus phylogeny with indication of their AL-proximity.

The lupine tRNA-Gly^GCC^ of Figure 1B has the same loops as 1200 tRNAs of different species extracted from the tRNAviz database (cf. [12] and Supplementary Material Table S2) containing the same AL sequence of nucleotides. This remarkable invariance of tRNA loops had already been noted by Manfred Eigen [13] and is related to the following variational problem [14]. Is there a circular RNA of minimum length comprising codons from the 20 synonymy classes of the genetic code, with a possible overlap? The answer is negative for lengths 20 and 21. For length 22, there are 29,520 solutions among the 10^22^ possible, if we authorize a supplementary codon from the STOP class (Figure 2). Of these 29,520 solutions, 1280 start with AUG, end with a STOP codon and have a codon in all 20 amino acid classes, including twice AUG (Rule R). If we look for solutions having, in addition to the circular shape, a second hairpin shape of maximum enthalpy (requiring a complementarity of two halves of the loop), we find 25 solutions (Rule R*), including 10 from the 1280 previous solutions (Rule R**), and their barycenter is the sequence, which has either a functional circular form (capable of catalyzing peptide synthesis) or a stable hairpin form, with the two coexisting in equilibrium (Figure 3 and Supplementary Material Figure S1).

### 2.3. Traces of the Archetypal Loop AL in the Present Genomes

If AL could have played a role in the construction of the first peptides, analogous to the role of the ribosome in current protein synthesis, we should find traces of it in the RNAs linked to ribosomal function. We have already seen its involvement, through four fragments, in the loops of the tRNA-Gly linked to the GCC anticodon. More generally, we find the succession of AL nucleotides (or of their family, with Y designating puric acids C and U, and R pyrimidine acids A and G) in the loops of numerous tRNAs (see Supplementary Material Table S2) and in numerous genomes of giant viruses [15] or viroids [16] (Figure 3).

**Figure 4:**
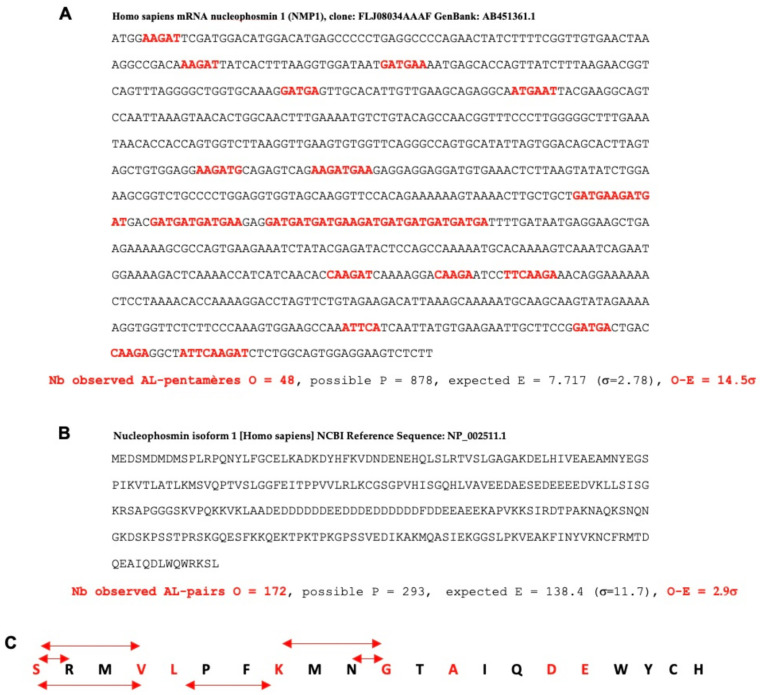
(**A**) AL-proximity of human nucleophosmin 1. (**B**) Same calculation for AL-pairs. (**C**) Most frequent AL-pairs observed (linked by two red arrows) and comparison with the oldest amino acids in the genetic code according to Koonin’s [22] and Trifonov’s [23] orders.

The proximity to AL (or AL proximity) noted in Figure 3 around the circular tree of giant viruses is calculated in the following way: we count the number O of common pentamers between the nine pentamers of the head of the hairpin form of AL, the easiest to fragment, called AL-pentamers {ATTCA, TTCAA, TCAAG, CAAGA, AAGAT, AGATG, GATGA, AATGA, ATGAA, TGAAT} and those of the studied RNA sequence of length n. Then, we calculate the expected number E, equal to the possible number P = n-4, multiplied by the probability p = 9/1024 of observing one of the nine AL-pentamers. The standard deviation of E is equal to σ = (Pp(1-p))^1/2^, and the AL-proximity equals (O-E)/σ. Figure 4A shows such a calculation for the human nucleophosmin 1 mRNA, involved in the construction of the ribosome. Its AL-proximity (14.5σ) is very high, because the probability of such a difference between observed and expected pentamers is of the order of 10^−14^ [17,18,19,20,21]. Figure 4B shows an identical calculation for a proximity between pairs of the sequence of amino acids corresponding to the succession of AL codons at a distance of at most 12 nucleotides from a given codon (called AL-pairs) and pairs of successive amino acids in the studied protein. The number of AL-pairs is significant (the probability of the difference between observed and expected pairs equal to 10^−3^) and the most frequent pairs are 9-12 nucleotides apart, corresponding to an efficient catalysis of the di-peptide biosynthesis by AL (Figure 4C).

**Figure 5:**
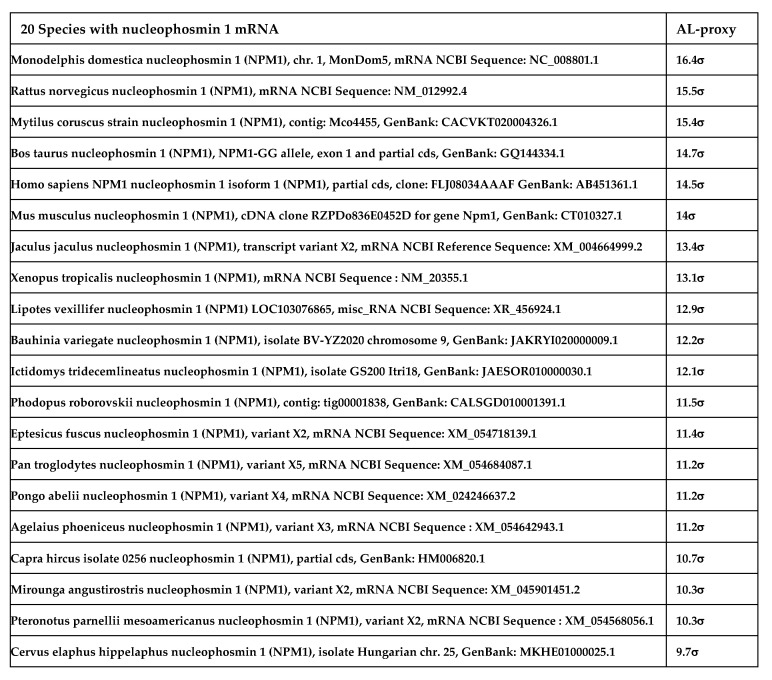
AL-proximity of nucleophosmin 1 (NPM1) mRNAs in 20 species of eukaryotes.

The abundance of traces of AL in nucleophosmin 1 is not limited to human species, and Figure 5 shows 20 species of eukaryotes having a nucleophosmin 1 mRNA close to AL, in terms of their content of AL-pentamers. The same is true for many RNAs and proteins (like nucleolin, see Supplementary Material Table S3) involved in the construction and functioning of the ribosome in its current version in eukaryotes, reinforcing the hypothesis of an ancient protein construction mechanism involving AL.

### 2.4. Ribosomal Proteins and rRNA Components of the Current Ribosomes

**Figure 6:**
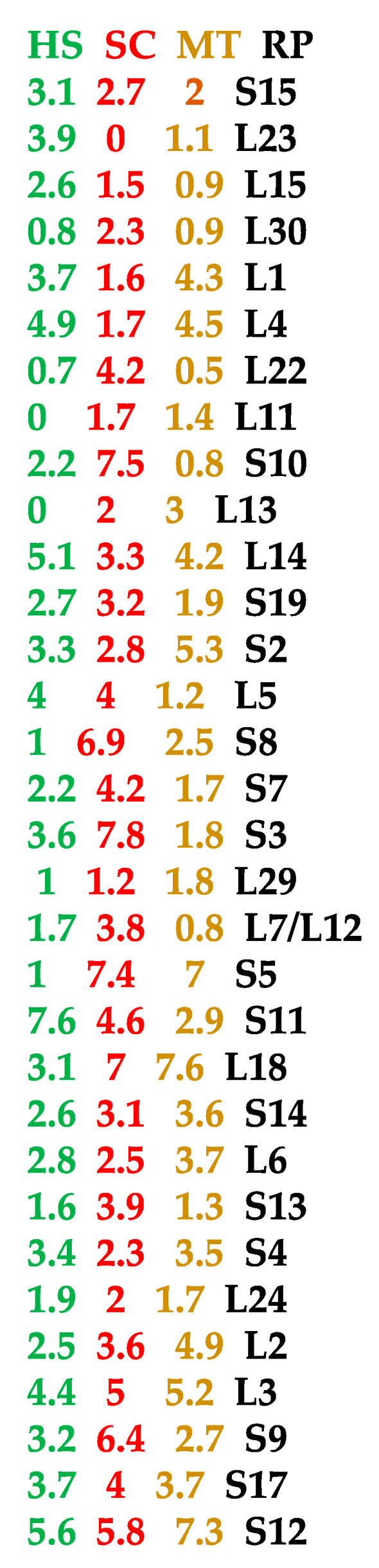
AL-proximity of ribosomal RNA and proteins (RP) from Homo sapiens (HS, in green), Saccharomyces cerevisiae (SC, in red) and Marine Group I thaumarchaeote YK1309 (MT, in brown) listed from the earliest (top) to the oldest (bottom) during evolution [24].

In order to strengthen the hypothesis that the AL ring is an ancient structure, we will calculate the AL-proximity of the ribosomal proteins (RPs) ordered following their anteriority. In Figure 6, the mean M_o_ (resp. M_n_) of AL-pentamer proximities for the 11 most ancient (respectively, recent) ribosomal proteins (after Gustavo Caetano-Anollés in [24]) is equal to 3.81 (respectively, 2.4). By applying a *t*-test of comparison of means, the 11 most ancient ribosomal proteins are closer to AL than the 11 earliest ones (*p* = 0.0001). This result is coherent with the hypothesis according to which AL is an ancient structure having had a role similar to that played by the current ribosome in multicellular eukaryotic species such as Homo sapiens and unicellular species such as Saccharomyces cerevisiae or in unicellular archaea such as Marine Group I thaumarchaeote YK1309.

## 3. Results

### 3.1. Phylogenetic Tree of Archaea

**Figure 7:**
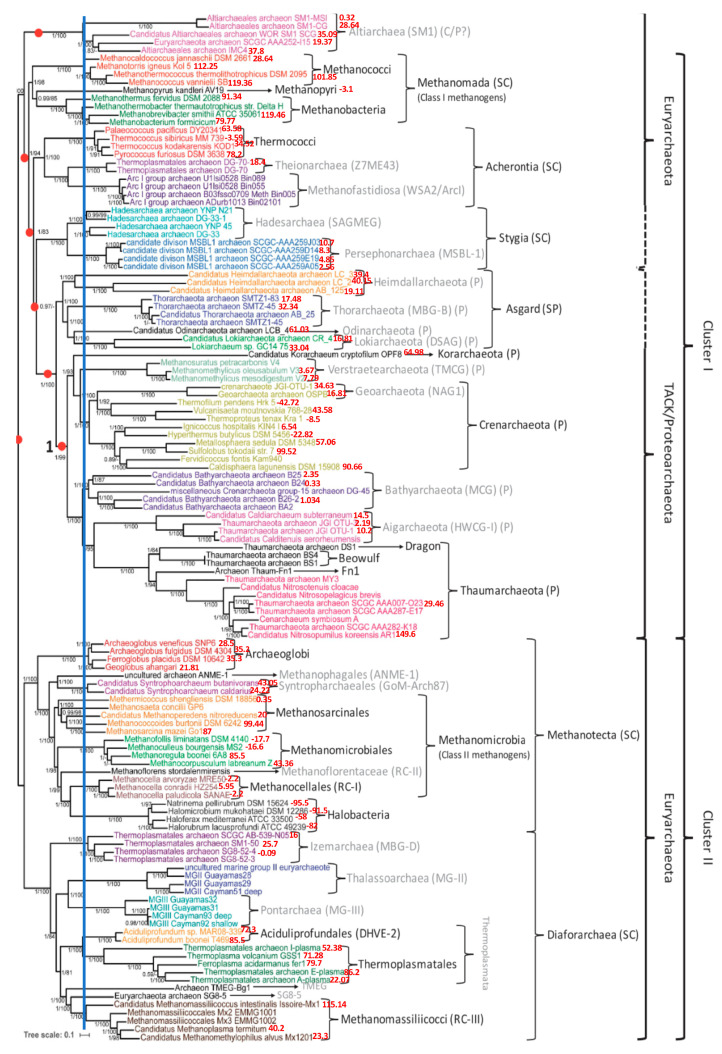
Phylogeny of 85 species of archaea obtained in [25] from 41 genes representing 8710 amino acid positions. The tree scale bar corresponds to the average number of substitutions per site, and node fractions refer to posterior probabilities calculated using the IQTree algorithm. The 41 genes consist of 36 genes from the Phylosift marker gene list, 2 RNA polymerase subunits A and B, and 3 universal ribosomal proteins (L7-L12, L30, S4). The taxonomic status of species families follows the following coding: C = class; P = phylum; SC = super class; SP = super phylum. The blue vertical line corresponds to the start node of both the Altiarchaea and Methanomassiliicocci sub-trees.

From the Archaeota phylum, 85 species ([25,26]) were classified through a classic phylogenetic tree using the IQTree algorithm by comparing 41 genes, including 3 coding for mitochondrial proteins, 2 for RNA-polymerase sub-units and 36 belonging to a gene list involved in the ribosomal architecture (S2, S3, S5, S7, S8, S9, S10, S11, S12/S23, S13e, S15, S19, S17, L1, L2, L3, L4/L1, L5, L6, L10, L11, L13, L14b/L23e, L15, L16/L10e, L18/L5e, L22, L24, L25/L23, L29). We added to this tree values (in red) of the AL-proximity of the entire genomes of the species concerned. These values are often in agreement with seniority in the tree. This is the case for the example of the Euryarchaeota phylum, which generally has a high value of AL-proximity (see Figure 7). 

On the other hand, certain species, such as Halobacteria, have negative values of their AL-proximity. This may be due to the presence of viral infections in these species, which have caused the negative distance of their genome to AL, proposed as the initial sequence [27]. 

### 3.2. Classification of Archaea by Maxwell 

**Figure 8:**
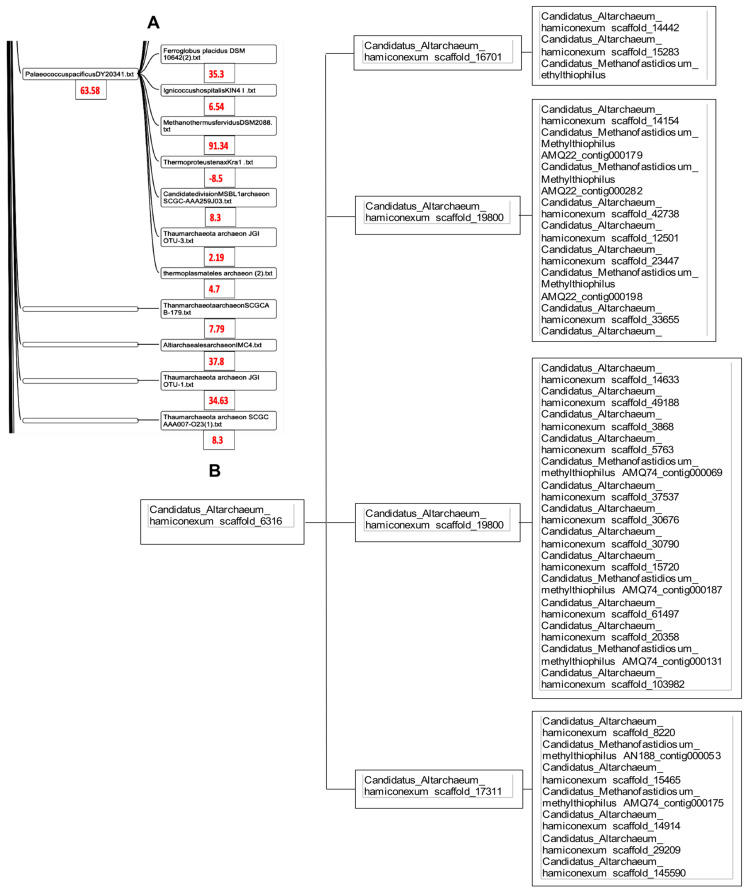
(**A**) Phylogenetic tree obtained using Maxwell© from complete genomes of the indicated species. (**B**) Part of this tree concerning the phylum Altarchaeota.

The classifier Maxwell is capable of processing the entire genomes of the 85 species of archaea from [25] in a few seconds. Only a small group of the clusters in Figure 8 corresponds to the phylum Altarchaeota, the rest being given in the Supplementary Material Table S4.

## 4. Discussion

We added to the tree in Figure 7 and Figure 8 the values (in red) of the AL-proximity of the entire genomes of the species concerned. We see that often, these values are in agreement with the ancient character of species in the tree. This is the case, for example, for the super class Methanomada (at the top of Figure 7), considered as one of the most ancient classes of archaea, whose species have a high value of AL-proximity. On the other hand, we have already remarked that certain species, such as Halobacteria or Methanomicrobiales, have negative values of AL-proximity. This may be due to the frequent viral infections in these species, distancing their genomes from the initial RNA ring AL [27,28]. For example, among the numerous viruses infecting these extremely halophilic archaea, there are many morphotypes (round, caudo, pleomorphic and spindle-shaped viruses) with a genome far from AL. This is, for example, the case for the Halogeometricum pleomorphic virus, whose AL-proximity is negative (−3.7σ) and which was thus able to contribute, via insertion of all or part of its genome, to the negativity of the Halobacteria genomes.

**Figure 9:**
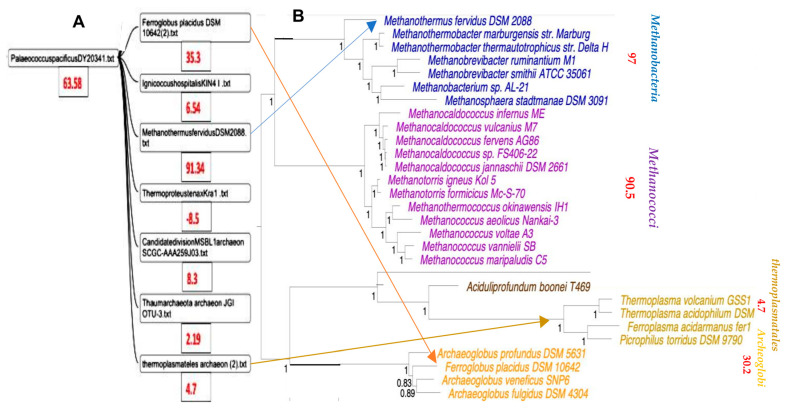
(**A**) Phylogenetic tree by Maxwell©. (**B**) Part of the archaea phylogenetic tree from [30] with mean AL-proximities (in red).

Regarding the phylogenetic tree obtained using Maxwell©, we see in Figure 8 that this classifier has brought together the different genomes of Altarchaeum hamiconexum (from the phylum Altarchaeota) with the genome of Methanofastidiosum methylthiophilus (from the class Methanomassiliicocci) well, which are on opposite sides of the phylogenetic tree in Figure 7. The reason is simple: these two classes are at the same distance from the tree root, because the same blue vertical line in Figure 7 corresponds to the start nodes of both their classes, Altiarchaeota and Methanomassiliicocci. There are numerous other phylogenetic trees for the archaea domain [29,30,31,32]. They have all the same general architecture analog to those in Figure 7. The coherence with the Maxwell^©^ architecture is not complete, but Figure 9 shows both an adequation of the AL-proximity order with the classification resulting from the phylogenetic tree given in [30] and a partial coherence with the Maxwell^©^ tree obtained by using only the raw sequence of the nucleotides of the complete genome of the concerned species from [25]. 

Numerous such examples of rapprochement in the same cluster can be found in Supplementary Material Table S4, and we will continue to multiply the species, by looking for the traces of AL in even more species of archaea, bacteria and eukaryotes, in order to strengthen the hypothesis of the “emergence of an RNA world, defined by RNA molecules with catalytic and replicative properties” [32] already published by Manfred Eigen [13].

## 5. Methodology

### 5.1. The Burrows–Wheeler Transform

The Burrows–Wheeler transform (BWT) is a lossless compression algorithm that rearranges strings into runs of similar characters in a reversible way [33,34]. When combined with a run-length encoding (RLE) algorithm, it yields a function that can be used in the computation of “Normalized Compression Distance” (NCD) or Vitányi distance, enabling the detection of similarities between information sequences such as repeated motifs, common deletion or insertions and other evolutionary patterns. BWT can be considered as a simplified compression algorithm with regard to more sophisticated ones commonly used in information theory applications, which can be lossless [35] or lossy [36]. Its main advantage over them is mathematically retrieving the symmetry of NCD. Such a property is essential to compare the genomic sequences of multiple species having coevolved under the action of the same operators. In the context of evolution, this indeed allows us to consider all of the eleven different genomic established operators, namely crossing over, mutation, translocation, insertion, deletion, transposition, inversion, repetition, symmetrization, palindromization and circular permutation. When these operators have been used with the same frequency during evolution, the Burrows–Wheeler transform is useful to compress the sequences of nucleotides coming from the same origin and having a similar evolutionary history.

### 5.2. Principles of the Burrows–Wheeler Transform (BWT)

The BWT algorithm transforms a string S of n characters by considering its n circular permutations (cyclic shifts), listing them lexicographically and retaining only the last character of each permutation. A final string F = BWT(S) is formed from these characters, where the ith character of F is the last character of the ith permutation. In addition to F, the BWT algorithm computes the rank s of the original string S in the list of permutations, and it is possible to integrally reconstruct S from F plus rank s. The run-length encoding of the string F, denoted RLE(F), is eventually obtained by replacing any sub-sequence of F repeating the same character, like TTT, by the number of repetitions followed by the repeated character, here 3T. Hence, the format of the final expression of the RLE(BWT(S)) is made first of the rank s, then of the characters of BWT(S) with an ultimate simplification: if a character C is met n(C) consecutive times, the final sequence is, after s, made of the succession of the characters C of BWT(S) preceded by their number n(C) of repetitions.

### 5.3. “Normalized Compression Distance” (NCD) or Vitányi Distance

**Figure 10:**
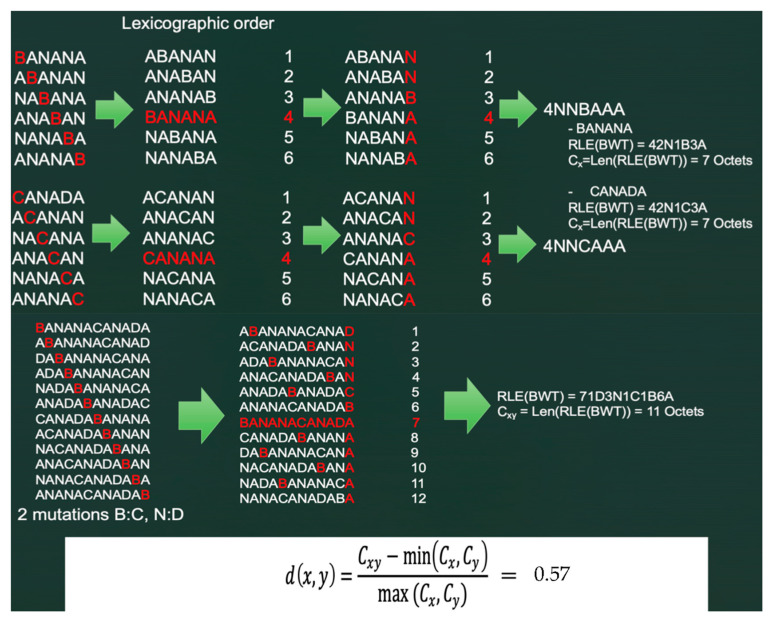
Burrows–Wheeler transform (BWT) of two words BANANA and CANADA, with two mutations B:C and N:D. Run-lengths (RLEs) of BWT transforms of BANANA, CANADA and concatenation BANANACANADA have, respectively, 7 and 11 Octets, and their NCD distance equals 0.57.

The Vitányi distance is a measure used to quantify the similarity between two sequences, x and y, based on their compressed representation [37,38]. Information compression is used here as a proxy for similarity. The intuition that stands behind this approach is that similar sequences can be compressed with the same efficiency, in contrast to dissimilar ones. In the context of Maxwell© functioning, this consists of calculating for x, y and the concatenated word xy the length of the RLE version of their Burrows–Wheeler transform (BWT), that is, respectively, if Len[string] denotes the string length, the values of the coefficients Cx = Len[RLE(BWT(x)], Cy = Len[RLE(BWT(y)] and Cxy = Len[RLE(BWT(xy)], and then calculating the ratio (Figure 10):

d(x,y) = [Cxy − min(Cx,Cy)]/max(Cx,Cy) (1)

At this stage, the NCD between x and y is the ratio between the size of the compressed representation of the concatenated sequence Cxy minus min(Cx,Cy) and the maximum of size of the compressed representations of each sequence individually, max (Cx,Cy).

The NCD or Vitányi distance is a real mathematical distance, with d(x,x) = 0, d(x,y) = d(y,x), and satisfies the triangular inequality d(x,z) ≤ d(x,y) + d(y,z).

Consider now the two words BANANA and CANADA and calculate the Vitányi distance between them (Figure 10). This Vitányi distance using the Burrows–Wheeler transform and run-length compression equals 0.57 (see https://gitlab.com/Orange-OpenSource/documentare/for the calculation program, accessed on 15 October 2023). 

### 5.4. Steps of the Maxwell© Algorithm of Clustering (MAC)

The principle of the Maxwell^©^ used to classify words belonging to the set {x_i_}_i=1,n_ is to constitute clusters from the distance matrix D_ij_ = d(x_i_,x_j_). The process is a dynamic and tessellation-based variant of the tree-based clustering approach proposed originally in [38]. In the Maxwell^©^ version, each triplet of words (x,y,z) constitutes a triangle in the graph associated with D, and the area A of this triangle is calculated using the classical Héron formula:

A = [p(p − a)(p − b)(p − c)]1/2,(2)

with a = d(x,y), b = d(y,z), c = d(z,x) and p = (a + b + c)/2.

The learning procedure can hence follow two strategies:

(1) *One-shot learning*. This procedure is static. It includes a unique forward pass whose role is to calculate the similarity clusters.
(2) *Active learning*. This procedure is dynamic. It includes one or several cycles of forward–backward passes. A backward pass consists of adjusting the validity of the clusters calculated previously in the forward pass based on a feedback loop. Feedback can be carried out either through automated decision, based on a validity calculation, or through the intervention of a human expert decision. At the end of a forward–backward cycle, invalidated data and/or clusters are rejected as singleton to possibly be then incorporated into a new forward–backward learning cycle, which can be summarized as follows:

(1) *Forward pass*
- The forward pass is the core of the original algorithm of Maxwell^©^. It takes as input the distance matrix D_ij_ and the triangulation standard deviation parameter σ to then execute the following steps:
- Triangulation property calculation: calculation of mean and standard deviation on histograms of triangle areas for filtering “large and deformed triangles” considered as outliers of the empirical distribution, according to the observed number of standard deviations σ from the area mean value retained;
- First-of-first (1-1) neighborhood pruning: examining sub-graphs whose “useless” (respectively, “best”) representative edges are identified as attached to the least (respectively, the most) connected nodes and remove (respectively, keep) them as central nodes;
- Local minima detection: processing subgraphs with several local minima, i.e., nodes whose neighborhood does not contain another node that is closer to the subgraph than the node itself, by using Voronoï networking (as in [5]) for detecting internal boundaries. In practice, this step can be implemented using the Graphiz open API [39] by testing at the end for sub-graphs of which mean and standard deviation depend on local minima, until Graphviz no longer detects any internal boundary;
- Singleton formation: storage of all the elements rejected by this statistical calculation in the form of “singleton clusters”;
- Final recall: re-clustering the population of singletons to detect new clusters.
(2) *Backward pass*

In the case of active learning, human expert intervention is permitted to validate singletons after their calculation as to invalidate other cluster elements as a feedback decision. A new recall of the forward pass can be then processed after such a feedback decision.

### 5.5. Toward Auto-Correction in Maxwell© with Multisets Used for Best Representative Nodes

The auto-correction method was recently added to the Maxwell^©^ platform as an active learning process and has enriched rules of rejects and recalls in an automatized way. When determining the statistical values of a cluster (barycenter node coordinates and mean distance of each element to this node), Maxwell© computes which node is the best representative object of this cluster. In the case of inability to choose a unique node, it builds a “multiset node” containing all the best representative nodes and computes the distances between each element and this multiset (definition of a multiset node is described well in [38,40,41]).

This method allows us to include variability in the representativity of a cluster. For example, one can consider a set of genomes representing different individuals from the same species. Users can determine a threshold for the multiset size, meaning a limit of its variability. This threshold parameter also allows us to detect an excessive cluster growth. In the case of exceeding this threshold, the considered cluster is deleted, and all nodes of its content are distributed again in their nearest neighbor cluster in a new cycle of active learning. We are now experimenting with the use of a similar strategy for labelled object processing (not applicable here), i.e., in order to minimize clusters having several labelled objects related to multi-hypothesis semantic values, a multi-hypothesis cluster with too many nodes can be deleted, and its content will be reprocessed in a new clustering cycle.

## 6. Conclusions

Without any contextual data, the classifier Maxwell© is capable of clustering long, full-genome sequences of archaea (for example, 1.78 M bp for Thermofilum pendens) by exploiting only their internal repetitions of motifs (such as pentamers common with an ancestral RNA ring) that appeared during evolution through the effect of the classical genetic operators responsible for genetic variability: mutations, translocations, insertions, deletions, transpositions, inversions, repetitions, symmetrizations, palindromizations, permutations and crossings over. Species that have co-evolved in the same environment have tended to be subject to the action of the same operators, of which their genomes keep track, and Maxwell is able to find the corresponding motifs and cluster genomes in relation to their frequency of occurrence. The example of archaea must be reinforced by experiments with larger genomes from other areas of evolution (bacteria and eukaryotes). In Supplementary Material Figures S2 and S3, we give some examples of such classifications with complete nuclear or mitochondrial genomes of several species (viral, bacterial and mammiferous).

The Maxwell MAC technique requires no training and can be used as a primer step for classical classification tools (like deep learning methods) requiring a training data set or an initial unbiased reference clustering. We will experiment in the future with using Maxwell for labeled object processing (in the case of a preorganization of the knowledge) in order to minimize clusters with several labeled objects related to multi-hypothesis semantic values. In genetics, this labeling can come from previous knowledge on genomes of other classes of the same phylum than the genomes to be classified, or it can result from a previous classification of which we have only kept the barycenter of the classes to accelerate the grouping process of labeled objects. Maxwell’s applications are therefore potentially very numerous in genetics, but more generally in all biomedical fields providing numerous data that are difficult to interpret without an extensive prior syntactic and/or semantic grouping work. 

## Data Availability

Data are contained within the article and Supplementary Materials.

## Supplementary Materials

The supporting information can be downloaded at: https://www.mdpi.com/article/10.3390/ijms242216278/s1.
