# Supplementary material for: An Unsupervised Classifier for Whole-Genome Phylogenies, the Maxwell© Tool
Source: Int J Mol Sci. 2023 Nov 13;24(22):16278. doi: 10.3390/ijms242216278 (PMC10671764; doi:10.3390/ijms242216278)
Supplement: Supplementary file 1 [file ijms-24-16278-s001.zip › ijms-2666463-supplementary.pdf]

Table S1. List of the classical classifiers with their main characteristics.

- CalibratedClassifierCV(LinearDiscriminantAnalysis())`: Linear Discriminant Analysis (LDA) is a classifier with a linear decision boundary, generated by fitting class conditional densities to the data and using Bayes' rule. The model fits a Gaussian density to each class. `CalibratedClassifierCV` is a probability calibration with isotonic regression or sigmoid.
- `CalibratedClassifierCV(QuadraticDiscriminantAnalysis())`: Similar to LDA but Quadratic Discriminant Analysis (QDA) allows for the decision boundary to be quadratic.
- `DecisionTreeClassifier()`: A decision tree classifier builds a model in the form of a tree structure, making decisions based on feature values.
- `ExtraTreeClassifier()`: An extremely randomized tree classifier, this algorithm randomizes certain decisions and thresholds more than a regular decision tree, adding additional randomness to the model.
- `DummyClassifier()`: This is a classifier that makes predictions using simple rules, and is useful as a simple baseline to compare with other (real) classifiers.
- `RandomForestClassifier()`: A forest of randomized decision trees, often yielding highly accurate predictions.
- `BaggingClassifier()`: Bagging (Bootstrap Aggregating) is a way to decrease the variance of the prediction by generating additional data for training from dataset using combinations with repetitions to produce multisets of the same size as the original.
- `ExtraTreesClassifier()`: Extra Trees is like a Random Forest, in that it builds multiple trees and splits nodes using random subsets of features, but with two key differences: it does not bootstrap observations (meaning it samples without replacement), and nodes are split on random splits, not best splits.
- `AdaBoostClassifier()`: The core principle of AdaBoost is to fit a sequence of weak learners (i.e., models that are only slightly better than random guessing) on repeatedly modified versions of the data.
- `GradientBoostingClassifier()`: Gradient Boosting builds an additive model in a forward stage-wise fashion; it allows for the optimization of arbitrary differentiable loss functions.
- `LogisticRegression()`: Logistic regression, despite its name, is a linear model for classification rather than regression. It is also used in the last layer of Neural Network models.
- `SGDClassifier()`: Linear classifiers (SVM, logistic regression, etc.) with SGD training. SGD stands for Stochastic Gradient Descent: the gradient of the loss is estimated each sample at a time and the model is updated along the way with a decreasing strength schedule (aka learning rate).
- `RidgeClassifier()`: Classifier using Ridge regression. This classifier first converts the target values into {-1, 1} and then treats the problem as a regression task (multi-output regression in the multiclass case).
- `RidgeClassifierCV()`: Ridge classifier with built-in cross-validation.
- `PassiveAggressiveClassifier()`: Passive Aggressive algorithms are online learning algorithms. Such an algorithm remains passive for a correct classification outcome, and turns aggressive in the event of a miscalculation, updating and adjusting.
- `LogisticRegressionCV()`: Logistic regression CV (Cross-Validation) classifier.
- `Perceptron()`: The Perceptron is another simple classification algorithm suitable for large scale learning.
- `BernoulliNB()`: Naive Bayes classifier for multivariate Bernoulli models.
- `GaussianNB()`: Gaussian Naive Bayes (GaussianNB) classifier. Can perform online updates to model parameters via the `partial_fit` method (for details on algorithm used to update feature means and variance online, see Stanford CS tech report STAN-CS-79-773).
- `KNeighborsClassifier()`: Classifier implementing the k-nearest neighbors vote.
- `NearestCentroid()`: Nearest ,Centroid is a simple algorithm representing each class by the centroid of its members. It also has no parameters to choose, making it a good baseline classifier.

- `MLPClassifier()`: Multi-layer Perceptron classifier. This model optimizes the log-loss function using LBFGS or stochastic gradient descent.
- `LabelPropagation()`: Label Propagation classifier is a semi-supervised learning method that propagates labels from the labeled to the unlabeled data.
- `SVC(probability=True)`: C-Support Vector Classification. The implementation is based on libsvm. The fit time complexity is more than quadratic with the number of samples which makes it hard to scale to datasets with more than a couple of 10000 samples.
- `LinearSVC()`: Linear Support Vector Classification. Similar to SVC with parameter kernel='linear', but implemented in terms of liblinear rather than libsvm, so it has more flexibility in the choice of penalties and loss functions and should scale better to large numbers of samples.

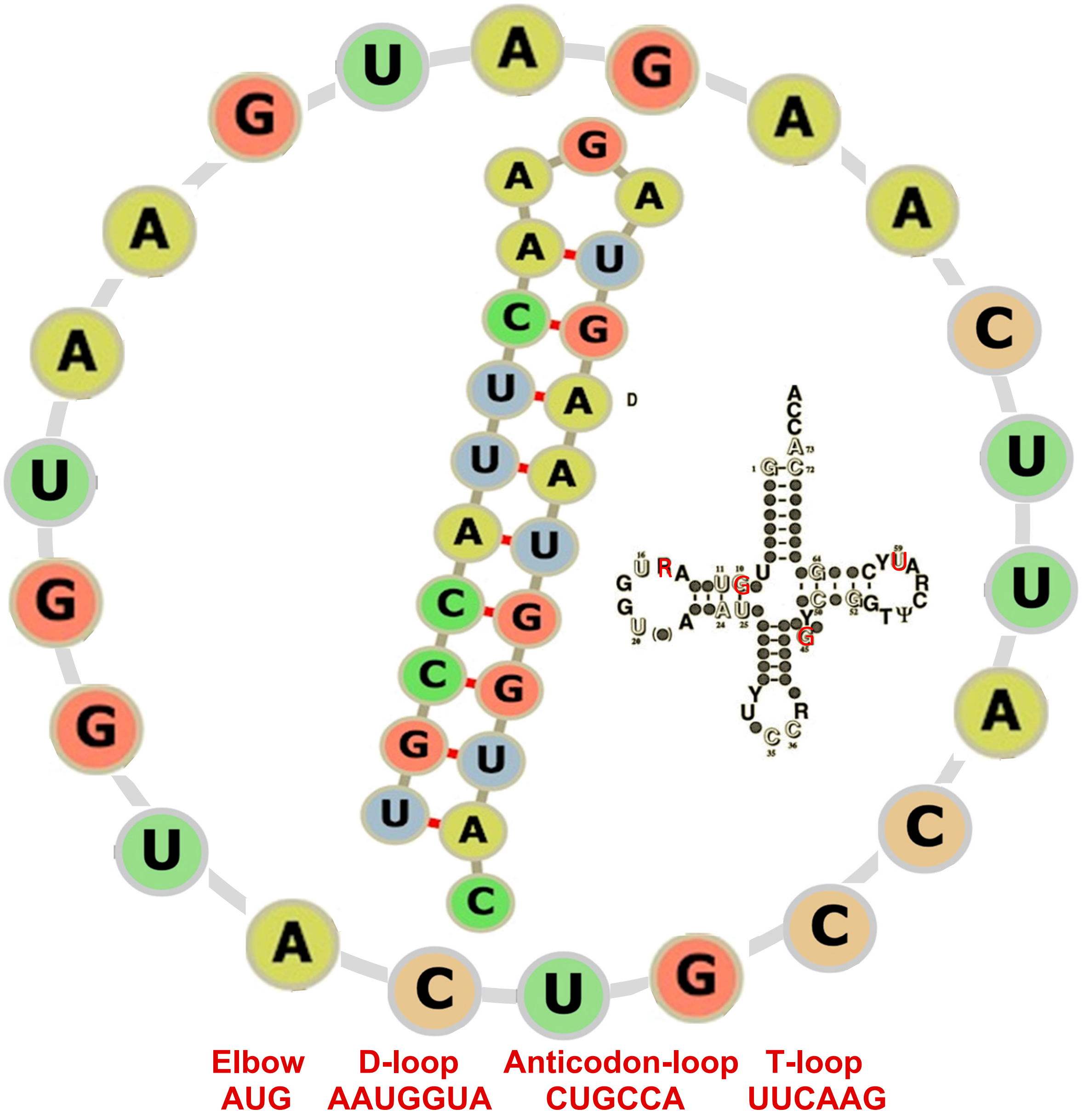

GCGGCUUUGAUGUAGACUGGUAUCAUACGGCCCUGCCACGGCCGACACCCGGGUUCAAAUCCCGGAGGCCGCACCA
Methanococcus maripaludis strain S2, complete sequence GenBank: BX950229.1: c1269120-1269048 tRNA-Gly

Figure S1. Top: Representation of the two forms (ring and inside hairpin) of the circular RNA AL (for Archetypal Loop) built from the most frequent nucleotides of the tRNA-Gly loops of

Archaeota. Inside the ring on the left of the hairpin, consensus tRNA-Gly secondary structure of yeast and mammalian tRNA-Gly's, with in red nucleotides different from the corresponding ones in the tRNA-GlyGCC of Methanococcus maripaludis; Bottom: tRNA-GlyGCC of M. maripaludis with AL-nucleotides in red.

Table S2. List of tRNA-Gly[GCC] from 246 species extracted from GtRNAdB (http://lowelab.ucsc.edu/GtRNAdb/) with indication (in red) of the pentamers shared with AL.

Cladorhizidae_sp_MBARI_D1340-BT2_Csp_MBARI_ tRNA-Gly-GCC-1-5 Sponge

GCACT**GATG**GTTC**A**G**TGGTA**GAATTCTCGC**CTGCCA**CGCGGGAGGCCCGGG**TTCAA**
TTCCCGGTCAGTGCA

Acetobacterium_woodii_DSM_1030_Acet_wood_DSM_1030_tRNA-Gly-GCC-1-2
Eubacteriaceae **(species)**

GCGGAAG**TG**GCTC**A**G**TGGTA**GAGCATCGCCT**TGCCA**AGGCGAGGGtCGCGAG**TTCA**
AATCTCGTCTTCCGCTCCA

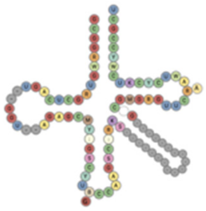

Acidobacterium_capsulatum_ATCC_51196_Acid_caps_ATCC_51196_tRNA-Gly-GCC-1-1 Acidobacteria (species)

GCGGGAG**T**AGCTC**A**G**TGGTA**GAGCATCGCCT**TGCCA**AGGCGAGGGtCGCGGG**TTCA**
AGTCCGTCTCCCGCTCCA

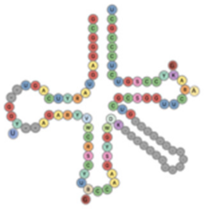

Aciduliprofundum_boonei_T469_Acid_boon_T469_tRNA-Gly-GCC-1-1 Euryarchaeota

GCGGGTG**TG**GTGTAGCC**TGGTA**ACACGAGGCC**CTGCCA**CGGCTTTGcCCCGGG**TTC**
AAATCCCGGCACCCGCA

Agrobacterium_fabrum_str_C58_Agro_fabr_C58_tRNA-Gly-GCC-1-1 Proteobacteria (species)

GCGGGTG**T**AGCTC**A**GG**GGTA**GAGCACAACCT**TGCCA**AGGTTGGGGtCGAGGG**TTCA**
AATCCCTTCGCCCGCTCCA

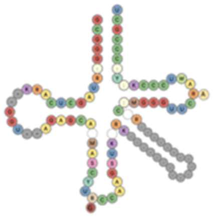

Alkaliphilus_metalliredigens_QYMF_Alka_meta_QYMF_tRNA-Gly-GCC-1-1 Clostridiaceae bacteria (species)

GCGGAAGTGGCTCAGTGGTAGAGCATCGCCTTGCCAAGGCGAGGGtCGCGAGTTCAAATCTCGTCTTCCGCTCCA

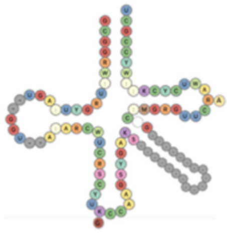

Alligator_mississippiensis_Amiss4_tRNA-Gly-GCC-4-2 Reptilia

GCACTGGTGGTTCAATGGTAGAATTCCTGCTTGCCATGCAGGGGaCCTGGGTTCAATTCCCAGCCAGTGCA

Aminobacterium_colombiense_DSM_12261_Amin_colo_DSM_12261_tRNA-Gly-GCC-1-1 Synergistota bacteria (species)

GCGGAAGTAGCTCAGGGGTAGAGCACAACCTTGCCAAGGTTGGGGtCGCGGGTTCAAATCCCGTCTTCCGCTCCA

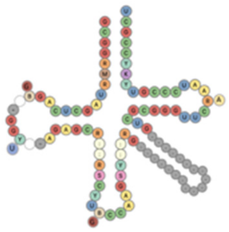

Anaerobaculum_mobile_DSM_13181_Anae_mobi_DSM_13181_tRNA-Gly-GCC-1-1
GCGGAAGTAGCTCAGGGGTAGAGCACCACCTTGCCAAGGTGGGGGcCGCGGGTTCAAATCCCGTCTTCCGCTCCA

Anaeromyxobacter_dehalogenans_2CP-1_Anae_deha_2CP_1_tRNA-Gly-GCC-1-1
GCGGGAATAGCTCAGTGGTAGAGCATCGCCTTGCCAAGGCGAGGGtCGAGGGTTCAAATCCCTTTTCCCGCTCCA

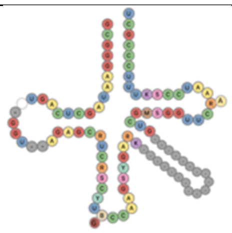

Arabidopsis_thaliana_Athal10_tRNA-Gly-GCC-3-1 Plant (species)

GCACCAG**TG**GTCT**A**G**TGGTA**GAATAGTACC**CTGCCA**CGGTACAGaCCCGGG**TTCAA**TTCCCGGCTGGTG
CA

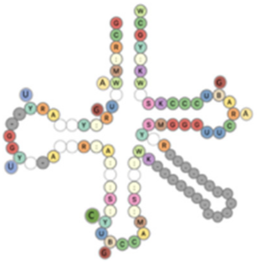

Archaeoglobus_fulgidus_DSM_4304_Arch_fulg_DSM_4304_tRNA-Gly-GCC-1-1
Euryarchaeota **(species)**

GCGCCG**ATG**GTGTAGCC**TGGTA**ACACAGGGGCTTGCCGAGCCC**CTGCC**CCGGG**TTC
AA**ATCCCGGTCGGCGCA

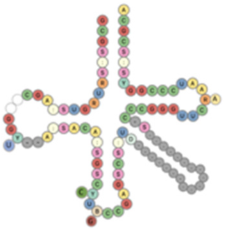

**Ascocoryne_sarcoides_NRRL_50072_Asco_sarc_NRRL_50072_tRNA-Gly-GCC-1-1
Helotiaceae Fungus (species)**

GCGTCGG**TG**GTTT**A**G**TGGTA**AAATCCATCGT**TGCCA**taagaatTTCAAttc**TTCAAG**cTCG
ATGGGcCCCGGGTTC

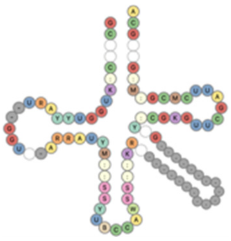

Azospirillum_sp_B510_Azos_B510_tRNA-Gly-GCC-2-1 Proteobacteria (genus)

GTATTTAAGCCAtTGGAAaATAACAATGTATTGCCAATTCGTAATCAgtAgGtCCGGGGT
TCAAATCCCTGTTGCG

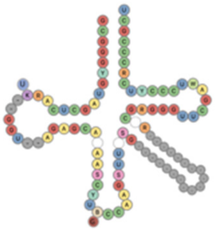

Balaenoptera_acutorostrata_scammoni_Bacut1_tRNA-Gly-GCC-766-1 Mammals (species)

GCATTGGTGGTTCAGTGGTAGAGTTCTTGCCTGCCATGTGGGAGGCCAGGGTTCAA
TTCCCCGCCAATGCA

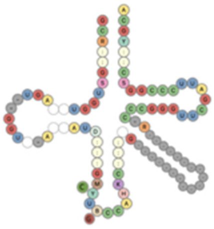

Batrachochytrium_dendrobatidis_JAM81_Batr_dend_JAM81_tRNA-Gly-GCC-1-2 Fungus (species)

GCGCTAGTGGTTTAGTGGTAAAATTCATCGTTGCCATCGATGAGcCCCCGGTTCAATT
CCGGGCTAGCGCA

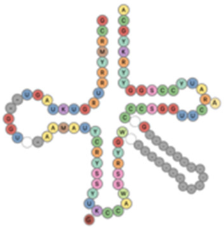

Bipolaris_sorokiniana_ND90Pr_Bipo_soro_ND90Pr_tRNA-Gly-GCC-1-3 Fungus (species)

CATCGG**TG**GTTT**A**G**TGGTA**AAATTAGCCGT**TGCCA**tccactctggtcc**TTCAA**aggtcccagctgg acaacgTCGGCT

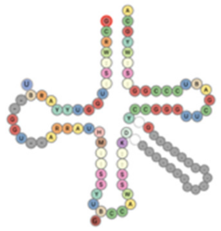

Blattabacterium_sp_Blaberus_giganteus_BGIGA_Blat_Blaberus_giganteus_BGIGA_ tRNA-Gly-GCC-1-1 Bacteroidetes (genus)

GCGAGA**AT**AGCTCAGT**TGGTA**GAGCACGACCT**TGCCA**AGGTCGGGGcCGCGGG**TTC AA**ATCCCGTTTCTCGCT

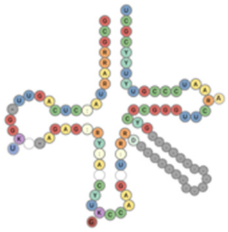

Blochmannia_endosymbiont_of_Camponotus_Colobopsis_obliquus_757_ tRNA-Gly-GCC-1-1 Gammaproteobacteria (species)

GCGGGA**AT**AGCTCAGT**TGGTA**GAGTACAACCT**TGCCA**AGGTTGGGGtCGCGAG**TTCA AG**TCTCGTTTCCCGCT

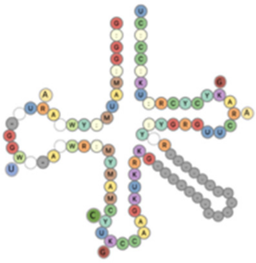

Blumeria_graminis_f_sp_tritici_96224_Blum_gram_f_tritici_96224_tRNA-Gly-GCC-7-1 Fungus (species)

GGTTGGA**TG**GTGCGG**TGGTA**cATCATcttGGCTGgT**CTGCCA**agtagagtctggatagcttggaca catatcccactcgAGaCAGTAGAtCGCTGG**TTCAA**ATCCAGC

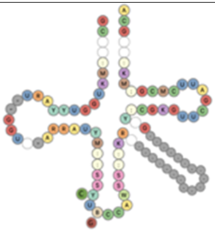

Borrelia_chilensis_VA1_Borr_chil_VA1_tRNA-Gly-GCC-1-1 Spirochaetaceae bacteria

GCGAAAG**T**AACTC**A**GG**GGTA**GAGTGTCACCT**TGCCA**AGGTGAAAGtCGCGGG**TTCAA**ATCCCGTCTTTCGCT

Bos_taurus_Btaur9_tRNA-Gly-GCC-3224-1 Mammals (assembly)

TCCTAGG**TG**GCTC**AATGGTA**AAAATACTTGC**CTGCCA**aAGCAGGAGaCTCAGG**TTCAA**TCCCTGGTCCAGGAA

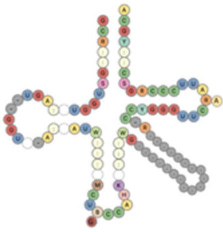

Botrytis_cinerea_B0510_Botr_cine_B05_10_tRNA-Gly-GCC-1-8 Fungus (species)

GCGTTTG**TG**GTTT**A**G**TGGTA**AAATCCATCGT**TGCCA**tctatctcttcttga**TTCAAG**aagagtgagaaacaac

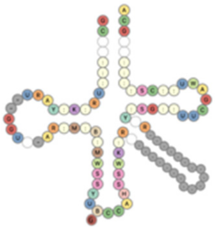

Branchiostoma_floridae_Bflor2_tRNA-Gly-GCC-2-1 Chordata

GCATCGG**TG**GTTC**A**G**TGGTA**GAATTCTCGC**CTGCCA**CGCGGGAGGCCCGGG**TTCAA**TTCCCGGCCGATGCA

Buchnera_aphidicola_str_5A_Acyrthosiphon_pisum_Buch_aphi_5A_Acyrthosiphon_pisum_tRNA-Gly-GCC-1-1 Gammaproteobacteria (species)

GCGGGA**AT**AGCTCAGT**TGGTA**GAGCACAACCT**TGCCA**AGGTTGGGGtCGCGAG**TTCAAG**TCTCGTTTCCCGCTCCA

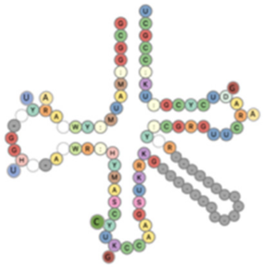

Caenorhabditis_brenneri_Cbren3_tRNA-Gly-GCC-5-1 Nematoda (assembly)

GCATCGG**TG**GTTC**A**G**TGGTA**GAATGCTCGC**CTGCCA**CGCGGGCTGCCCGGG**TTCAA**TTCCCGGTCGATGCA

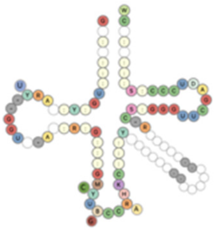

Caldivirga_maquilingensis_IC-167_Cald_maqu_IC_167_tRNA-Gly-GCC-1-1 Thermoproteota archaea (species)

GCGGCCG**T**AGTCTAGTC**tGGT**ttAGGATGGCGGC**CTGCC**GCGCCGCAGAtCCCGGG**TTCAA**ATCCCGGCGGCCGCA

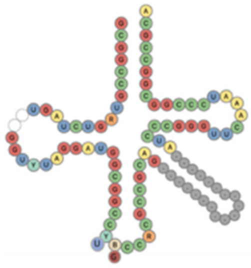

Callithrix_jacchus_Cjacc3_tRNA-Gly-GCC-4-1 Primates (species)

GCATGGG**TG**GTTC**A**G**TGGTA**GAATTCTCGC**CTGCCA**CGCGGGAGtCCTGGG**TTCAA**TCCCCGGCCCACGCA

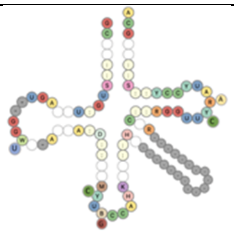

Calothrix_sp_PCC_7507_Calo_PCC_7507_tRNA-Gly-GCC-2-1 Cyanobacteria (genus)

GCGGATGTGGTGTAAGGGCAACACCGGAGTATGCCAAGCTCCAAaTGCGAGTTCAA
CTCTCGTCGTCCGCT

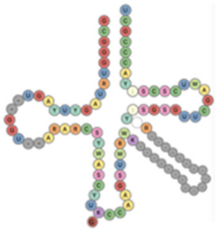

Campylobacter_jejuni_subsp_doylei_26997_Camp_jeju_doylei_269_97_tRNA-Gly-GCC-2-1 Campylobacterota (species)

GCGGGAATAGCTCAGGGGTAGAGCACAACCTTGCCAAGGTTGGGGtCGCGAGTTCA
AATCTCGCTAACCGCACCA

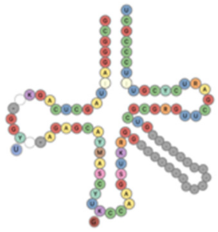

Candidatus_Azobacteroides_pseudotrichonymphae_genomovar_CFP2 _tRNA-Gly-GCC-1-1 Bacteroidetes

GCGAAAATAGCTCAGTTGGTAGAGTATAACCTTGCCAAGGTTAGGGtCGCGGGTTCA
AGTCCCGTTTTTCGCT

Candidatus_Baumannia_cicadellinicola_BGSS_Cand_Baum_cicadellinicola_BGSS_tRNA-Gly-GCC-1-1 Proteobacteria

GCGGGAATAGCTCAGTGGTAGAGCACAACCTTGCCAAGGTTGGGGtCGCGAGTTCA
AACCTCGTTTCCCGCTCCA

Candidatus_Blochmannia_chromaiodes_str_640_Cand_Bloc_chromaiodes_640_tRNA-Gly-GCC-1-1 Gammaproteobacteria (genus)

GCGGGAATAGCTCAGTTGGGAGAGCACAACCTTGCCAAGGTTGGGGtCGCGAGTTC
AAATCTCGTTTCCCGCTCCA

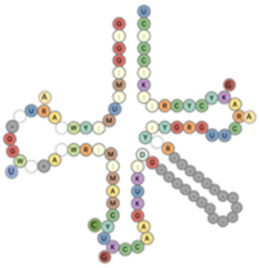

Candidatus_Caedibacter_acanthamoebae_Cand_Caed_acanthamoebae_tRNA-Gly-GCC-1-1 Gammaproteobacteria

GCGGGCGTAGCTCAGGGGTAGAGCACAACCTTGCCAAGGTTGGGGtCGAGGGTTCAAATCCCTTCGCCCGCTCCA

Candidatus_Caldiarchaeum_subterraneum_Cand_Cald_subterraneum_tRNA-Gly-GCC-1-1 Aigarchaeota

GCGGCGGTCGTCTAGCCTGGTAGGACACCAGCCTGCCACGCTGGGAGtCGCGGGTTCAAATCCCGCCCGCCGCA

Candidatus_Carsonella_ruddii_DC_Cand_Cars_ruddii_DC_tRNA-Gly-GCC-1-1 Gammaproteobacteria

GCGAAAGTATCTTAATGGTAAAGTATCACCTTGCCATGGTGAAAGtTGCGAGTTCAAGTCTCGTCTTTCGCT

Candidatus_Cloacimonas_acidaminovorans_str_Evry_Cand_Cloa_acidaminovorans_Evry_tRNA-Gly-GCC-2 Cloacimonetes bacteria

GCGGGAATAGCTCAGTTGGCAGAGCGCAACCTTGCCAAGGTTGAAGtCGCGGGTTCAAACCCCGTTTCCCGCTCCA

Candidatus_Endolissoclinum_faulkneri_L5_Cand_Endo_faulkneri_L5_tRNA-Gly-GCC-1-1 Alphaproteobacteria

GCGGGCGTAGCTCAGTGGTAGAGCACAACCTTGCCAAGGTTGGGGtCGTGAGTTCAAGTCTCATCGTCCGCTCCA

Candidatus_Korarchaeum_cryptofilum_OPF8_Cand_Kora_cryptofilum_OPF8_tRNA-Gly-GCC-1-1 Korarchaeota

GCGGCCGTAGTCTAGCCTGGAcAGGATGGGGGCCTGCCACGTCCCAGACCCGGGTTCAAATCCCGGCGGCCGCA

Candidatus_Koribacter_versatilis_Ellin345_Cand_Kori_versatilis_Ellin345_tRNA-Gly-GCC-1-1 Acidobacteriota

GCGGGAGTAACTCAGTGGTAGAGTGCGACCTTGCCAAGGTCGAAGtCGCGGGTTCAAATCCCGTCTCCCGCTCCA

Candidatus_Methylomirabilis_oxyfera_Cand_Meth_oxyfera_tRNA-Gly-GCC-1-1 Methylomirabilota bacteria

GCGGGA**AT**AGCTC**A**GC**GGTA**GAGCATCGCCT**TGCCA**AGGCGAGGGtCGCGGG**TTCA**
AATCCCGTTTCCCGCTCCA

Candidatus_Nasuia_deltocephalinicola_str_NAS-ALF_tRNA-Gly-GCC-1-1 Betaproteobacteria

GGGTAGC**T**T**AATGGTA**AAGCGTAACTT**TGCCA**AAGTTGAGAtTGCGAG**TTCAA**ATCTC
GTCCCTT

Candidatus_Sulcia_muelleri_CARI_Cand_Sulc_muelleri_CARI_tRNA-Gly-GCC-1-1 Bacteroidota

GCGAGA**AT**AGCTCATT**TGGTA**GAGTACTACCT**TGCCA**AGGTAG**TGGTA**GCGGG**TTCA**
AATCCCGTTTCTCGCT

Candidatus_Zinderia_insecticola_CARI_Cand_Zind_insecticola_CARI_tRNA-Gly-GCC-1-1 Betaproteobacteria

GCGAAAG**T**AGCTCA**A**T**TGGTA**GAGCAATACCT**TGCCA**AGGTATAGGtTGAGAG**TTCAA**
AACTCTTCTTTCGCT

Cavia_porcellus_Cporc3_tRNA-Gly-GCC-204-1 Mammals (species)

GcGTATC**T**C**A**G**TGGTA**GAATACTTGC**CTGCCA**TGCACAAAGcTCTGGG**TTCAA**TTCCT
AGCATtgCA

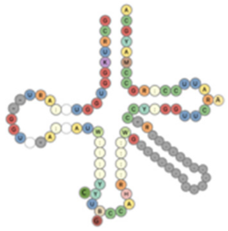

Ceratodon_purpureus_GG1_Cpurp_GG1_1_tRNA-Gly-GCC-9-1 Bryophyta

GGGtttT**ATG**TCT**A**G**TGGTA**GAATAGTACC**CTGCCA**CGGTACAGaCCCGGG**TTCAA**TTC
CCGGCCGGTGCA

Ceratodon_purpureus_R40_Cpurp_R40_1_tRNA-Gly-GCC-3-1 Bryophyta

GCGGAAA**T**AGCTT**AATGGTA**GAGTATAGCCT**TGCCA**AGGCTGAGGtTGAGGG**TTCAA**
GTCCCTTTTTCCGCT

Chara_braunii_S276_Cbrau1_tRNA-Gly-GCC-11-1 Viridiplantae

GTGGAA**AT**AGCTT**AATGGTA**GAGTATAGCCT**TGCCA**AGGCTAAGGtTGAGGG**TTCAA**
ATCCCTTTTTCCGCT

Chlamydia_muridarum_MopnTet14_Chla_muri_MopnTet14_tRNA-Gly-GCC-1-1 Chlamydiota bacteria (species)

GCGGGTG**T**AGCTC**A**G**TGGTA**GAGCGCCACGT**TGCCA**ACGTGAAGGtCGTGAG**TTCA AG**CCTCATCACCCGCT

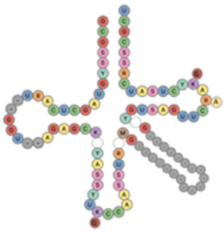

Chlamydophila_abortus_AB7_Chla_abor_AB7_tRNA-Gly-GCC-1-1 Chlamydiota bacteria
GCGGGTG**T**AGCTC**A**G**TGGTA**GAGCGCCACGT**TGCCA**ACGTGAAGGtCGTGAG**TTCA AG**CCTCATCACCCGCT
Choloepus_hoffmanni_Choff2_tRNA-Gly-GCC-2-1 Mammals

GCATTGG**TG**GTTC**A**G**TGGTA**GAATTCTCGC**CTGCCA**CGCGGGAGGCCCGGG**TTCAA** TTCCCGGCCAATGCA

Chrysemys_picta_bellii_Cpict2_tRNA-Gly-GCC-24-1 Reptilia turtle (species)

GCATTGG**TG**GTTC**A**G**TGGTA**GAACTCTCCC**CTGCCA**CGTGGGAGGCCTAGA**TTCAA**T TCCCAACCAGTATA

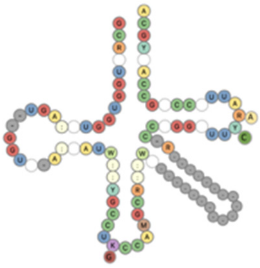

Cladorhizidae_sp_MBARI_D1340-BT2_Csp_MBARI_tRNA-Gly-GCC-1-1 Demospongiae

GCACTG**ATG**GTTC**A**G**TGGTA**GAATTCTCGC**CTGCCA**CGCGGGAGGCCCGGG**TTCAA** TTCCCGGTCAGTGCA

Closterium_sp_NIES-67_Clost1_tRNA-Gly-GCC-5-1 Charophyta

GTGCGCTG**T**TT**A**G**TGGTA**GAATAGCACC**CTGCCA**tgtgccCGGTGCAGaCCTGGG**TTCA A**TTCCCGGCTGGCACA

Clostridium_kluyveri_DSM_555_Clos_kluy_DSM_555_tRNA-Gly-GCC-2-1 Eubacteriales (species)

GCGGGAG**TG**GCTC**A**G**TGGTA**GAGCGTCACCT**TGCCA**AGGTGAACGtCGCGGG**TTCA A**ATCCCGTCTTCCGCTCCA

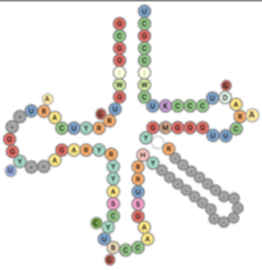

Corallococcus_coralloides_DSM_2259_Cora_cora_DSM_2259_tRNA-Gly-GCC-1-2 Myxococcales bacteria (species)

GCGGGAATAGCTCAGCGGTAGAGCATCGCCTTGCCAAGGCGAGGGtCGAGGGTTCA
A
ATCCCTTTTCCCGCTCCA

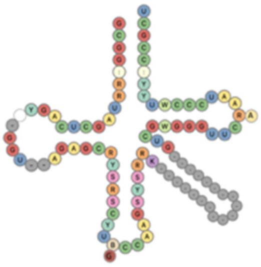

Cricetulus_griseus_Cgris1_tRNA-Gly-GCC-301-1 Mammals (halmster) (species)

TCTTGGTTGCAGCTCAGTGGTAAAGCATTTAGCTGCCATTTGCAAGGcTTTGGATTCA
ATCCCCAgGACTAAGAA

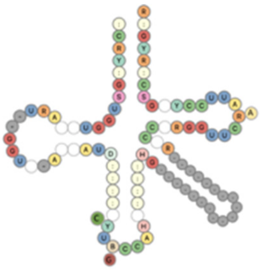

Curvularia_lunata_CX-3_Curv_luna_CX_3_tRNA-Gly-GCC-1-5 Fungus (species)

GCATCGGTGGTTTAGTGGTAAAATTAGCCGTTGCCATTCATCTGTTCAATTGATTCAC
TTCACAGAACAGCATGAG

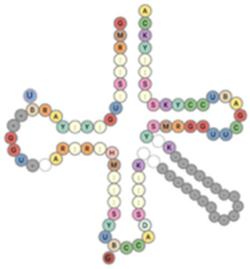

Cylindrospermum_stagnale_PCC_7417_Cyli_stag_PCC_7417_tRNA-Gly-GCC-2-1 Cyanobacteria (species)

GGAAGTGGTAGCTCAACTGGAAGAGCGCTCGACAGCCAATCGGGAGGtTGTGAGTTC
AAGTCTCACCCCTTCCA

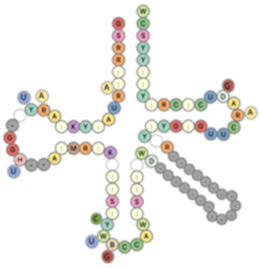

Dactylellina_haptotyla_CBS_20050_Dact_hapt_CBS_200_50_tRNA-Gly-GCC-2-1 Fungus (species)

GCGTTTG**TG**GTTT**A**G**TGGTA**AAATTCGTCGT**TGCCA**TTCAAtaatgataTCGACGAGcCCCGGG**TTCAA**TTCCCGG

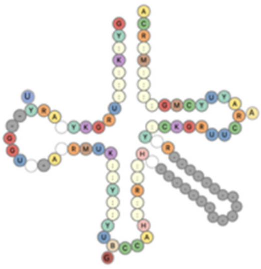

Danio_rerio_Dreri11_tRNA-Gly-GCC-15-1 Fish

GCATTGG**TG**GTTC**A**G**TGGTA**GAATTCTCGC**CTGCCA**CGCGGGAGaCCCGGG**TTCAA**TTCCCGGCTAATGCA

Dasypus_novemcinctus_Dnove3_tRNA-Gly-GCC-10902-1 Mammals (armadillo) (species)

GCaTGGG**TG**GTTC**A**G**TGGTA**GAATTCTCAC**CTGCCA**CACAGGAGGCCCAGG**TTCAAG**TCCCAGCCAAGCAC

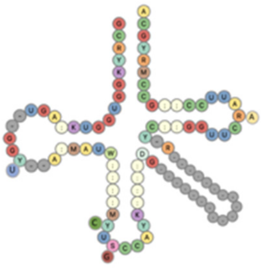

Desulfarculus_baarsii_DSM_2075_Desu_baar_DSM_2075_tRNA-Gly-GCC-1-1 Thermodesulfobacteriota (species)

GCGGGAG**T**AACTCAGAGGTAGAGTGCAACCT**TGCCA**AGGTTGAAGtCGCGGG**TTCAA**CTCCCGTCTCCCGCTCCA

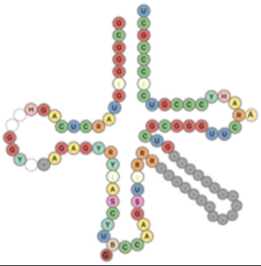

Desulfatibacillum_alkenivorans_AK-01_Desu_alke_AK_01_tRNA-Gly-GCC-1-1
Desulfatibacillum

GCGGGAATAACTCAGTGGTAGAGTGCGACCTTGCCAAGGTCGAAGtCGCGGGTTCA
AATCCCGTTTCCCGCTCCA

Desulfobacterium_autotrophicum_HRM2_Desu_auto_HRM2_tRNA-Gly-GCC-1-2
Thermodesulfobacteriota (species)

GCGGGAATAACTCAGTGGTAGAGTGCGACCTTGCCAAGGTCGAAGtCGCGGGTTCA
AATCCCGTTTCCCGCTCCA

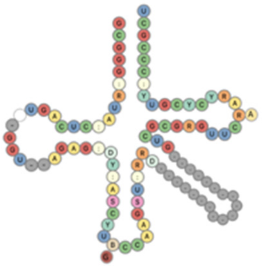

Thermodesulfobacteriota (species)

GCGGGAATAACTCAGTGGTAGAGTGCGACCTTGCCAAGGTCGAAGtCGCGGGTTCA
AATCCCGTTTCCCGCTCCA

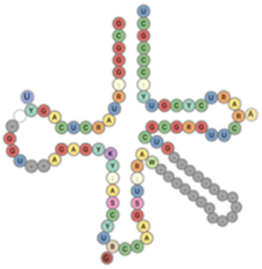

Desulfococcus_oleovorans_Hxd3_Desu_oleo_Hxd3_tRNA-Gly-GCC-1-1
Thermodesulfobacteriota (species)

GCGGGAATAACTCAGTGGTAGAGTGCGACCTTGCCAAGGTCGAAGtCGCGGGTTCA
AATCCCGTTTCCCGCTCCA

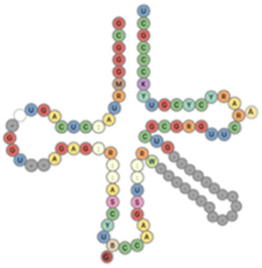

Desulfohalobium_retbaense_DSM_5692_Desu_retb_DSM_5692_tRNA-Gly-GCC-1-1
Thermodesulfobacteriota (species)

GCGGGAG**T**AACTCAG**TGGTA**GAGTGCAACCT**TGCCA**AGGTTGAAGtCGCGGG**TTCAA**
ATCCCGTCTCCCGCTCCA

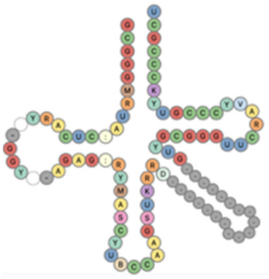

Desulfomicrobium_baculatum_DSM_4028_Desu_bacu_DSM_4028_tRNA-Gly-GCC-1-4 Desulfomicrobiaceae bacteria (species)

GCGGGAG**T**AACTCAG**TGGTA**GAGTGCAACCT**TGCCA**AGGTTGAAGtCGCGAG**TTCAA**
ATCTCGTTTCCCGCTCCA

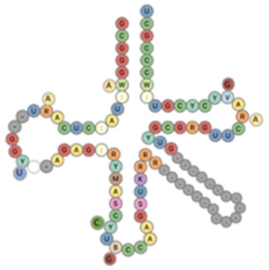

Desulfomonile_tiedjei_DSM_6799_Desu_tied_DSM_6799_tRNA-Gly-GCC-1-1 Desulfomonilaceae bacteria (species)

GCGGGA**AT**AACTCAG**TGGTA**GAGTGCAACCT**TGCCA**AGGTTGAAGtCGCGGG**TTCAA**
ATCCCGTTTCCCGCTCCA

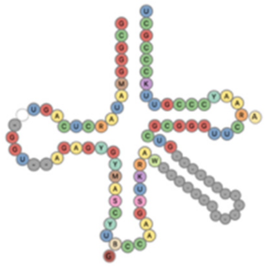

Desulfovibrio_africanus_str_Walvis_Bay_Desu_afri_Walvis_Bay_tRNA-Gly-GCC-1-1 Thermodesulfobacteriota (species)

GCGGGA**AT**AACTC**A**G**TGGTA**GAGTGCAACCT**TGCCA**AGGTTGAAGtCGCGGG**TTCAA**
ATCCCGTTTCCCGCTCCA

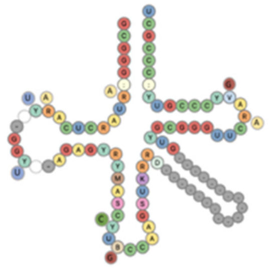

Desulfurella_acetivorans_A63_Desu_acet_A63_tRNA-Gly-GCC-1-1 Campylobacteriota (species)

GCGGGAATAGCTCAGTTGGTAGAGCACAACCTTGCCAAGGTTGGGGtCGCGGGTTC
AAGTCCCGTTTCCCGCTCCA

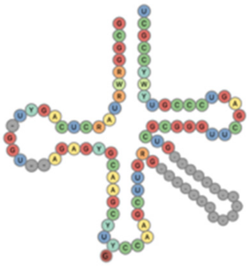

Desulfurobacterium_thermolithotrophum_DSM_11699_Desu_ther_DSM_11699_tRNA-Gly-GCC-2-1 Desulfurobacteriales (species)

GCGGGCGTAGCTCAGTTGGTAGAGCGAAACCTTGCCAAGGTTTAGGtCGCGGGTTCA
AGTCCCGTCGCCCGCTCCA

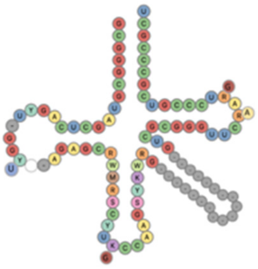

Dictyoglomus_thermophilum_Dict_ther_H_6_12_ATCC_35947_tRNA-Gly-GCC-1-1 Dictyoglomota bacteria (species)

GCGGGAGTAGCTCAGGGGTAGAGCGTCTCCTTGCCAAGGAGAAGGcCGCGGGTTCA
AATCCCGTCTCCCGCTCCA

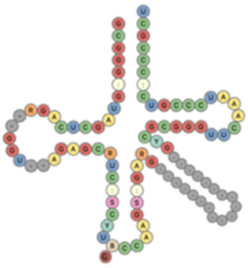

Dipodomys_ordii_Dordi2_tRNA-Gly-GCC-1-1 Mammals (rat) (species)

GCATGGGTGGTTCAGTGGTAGAATTTTTGCCTGCCACACAGGAGGCCTGGGTTCAAT
TTCCAGCCCATGAA

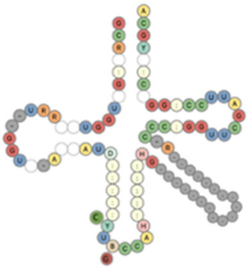

Dokdonia_sp_4H-3-7-5_Dokd_4H_3_7_5_tRNA-Gly-GCC-1-1 Bacteroidota

GCGAAAG**T**AGCTCAGGGGTAGAGCATCACCT**TGCCA**AGGTGGGGGtCGCGAG**TTCA**
AATCTCGTCTTTCGCT

Drechslerella_stenobrocha_248_Drec_sten_248_tRNA-Gly-GCC-1-1 Fungus (species)

GCGTTTG**TG**GTTTAG**TGGTA**AAATTCATCGT**TGCCA**t**CTGCC**tccgcaggtcaagTTCGATG
AGcCCCGGG**TTCAA**TTCCCGGCAGACGCA

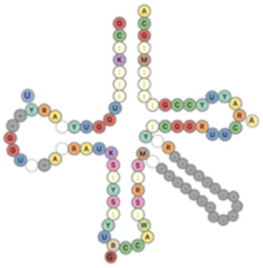

Echinops_telfairi_Etelf2_tRNA-Gly-GCC-394-1 Plant Asteraceae

CCCTGG**T**AGCACAG**TGGTA**ACAGCATTTGG**CTGCCA**TCCagAAAAGtCAGTGG**TTCAA**
ACCCACCagcctcTCAGTGGA

Erinaceus_europaeus_Eeuro2_tRNA-Gly-GCC-3-1 Mammals (hedgehog) (species)

ACATGGG**TG**GATCAG**TGGTA**GAATTCTTTC**CTGCCA**TGCGGGAGGCCCAGG**TTCAA**T
TCCCGGCTCATGCA

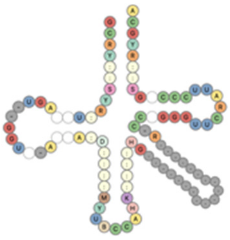

Erysipelothrix_rhusiopathiae_SY1027_Erys_rhus_SY1027_tRNA-Gly-GCC-1-1 Bacillota (species)

GCAGGTG**T**AGTTC**AATGGTA**GAACACGACCT**TGCCA**AGGTTGAGGCGGGGG**TTCAA**
TTCCCCTCACCTGCTCCA

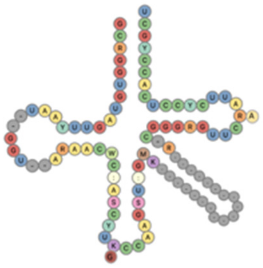

Eubacterium_acidaminophilum_DSM_3953_Euba_acid_DSM_3953_tRNA-Gly-GCC-1-1 Bacillota

GCGGAAG**TG**GCTCAG**TGGTA**GAGCATCGCCT**TGCCA**AGGCGAGGGtCGCGGG**TTCA**
AATCCCGTCTTCCGCTCCA

Exophiala_mesophila_CBS_40295_Exop_meso_CBS_40295_tRNA-Gly-GCC-1-1
Fungus (species)

GCATTGT**TG**GTTTAG**TGGTA**AAAT**CTGCC**GT**TGCCA**gtcttccttaacaggtctact**TTCAA**tgTC
GGCAGGcCCCGTGTTCGATTCACGGACAATGCA

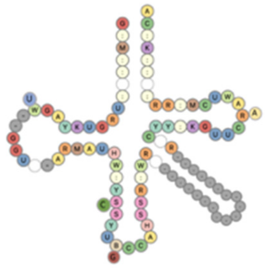

Faecalitalea_cylindroides_T2-87_Faec_cyli_T2_87_tRNA-Gly-GCC-1-1
Firmicutes bacteria (species)

GCAGATG**T**AGTTC**AATGGTA**GAACACAGCCT**TGCCA**AGGCTGATaCGGGGG**TTCAA**T
TCCCCTCATCTGCTCCA

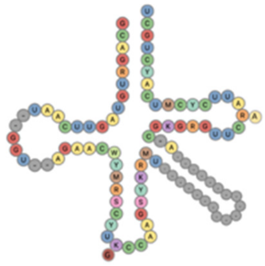

Fervidicoccus_fontis_Kam940_Ferv_font_Kam940_tRNA-Gly-GCC-1-1
Proteoarchaeota

GCGGCGG**T**CGTCTAGCC**TGG**AtcAGGACGCCGGC**CTGCCA**CGTCGGAAAtCCCGGG**T**
TCAAATCCCGGCCGCCGCA

Fistulina_hepatica_ATCC_64428_Fist_hepa_ATCC_64428_tRNA-Gly-GCC-2-1
Fungus (species)

GCATTA**ATG**GGGTAG**TGGTA**ACCTGGGTCGT**TGCCA**tagggattgagcacccacttgTCGACC
CGcCGGGGG**TTCAA**TTCCCCCTTAATGCA

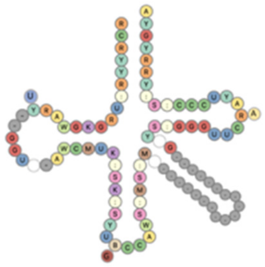

Formosa_agariphila_KMM_3901_Form_agar_KMM_3901_tRNA-Gly-GCC-1-1
Bacteroidota (species)

GCGAAAG**T**AGCTC**A**GG**GGTA**GAGCATCACCT**TGCCA**AGGTGAGGGtCGCGGG**TTCA**
AATCCCGTCTTTCGCT

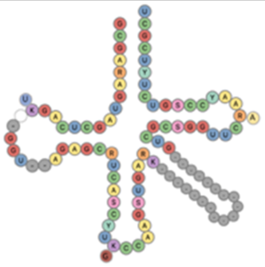

Geobacillus_sp_Y41MC1_Geob_Y4_1MC1_tRNA-Gly-GCC-3-1 Bacillota (genus)

GCGGAAG**T**AGTTC**A**G**TGGTA**GAACACCACCT**TGCCA**AGGTGGGGGtCGCGGG**TTCAAG**TCCCGTCTTCCGCT

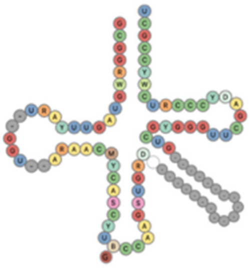

Geospiza_fortis_Gfort1_tRNA-Gly-GCC-1-1 Aves (species)

GCATTGG**TG**GTTC**A**G**TGGTA**GAATTCTCGC**CTGCCA**CGCAGGAGGCCAGGG**TTCAA**TTCCTGACCAAAGCA

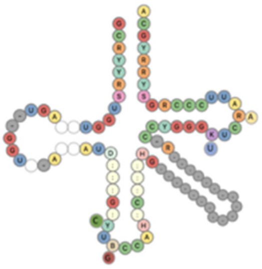

Glycine_max_Williams_82_Gmax2.1_tRNA-Gly-GCC-1-1 Plant (soybean) (species)

GTTGTTG**T**AGT**AT**A**GT**GATaAGTATTTTCCC**CTGCCA**CGGGAACGaTCCGGA**TTCAA**TCCCCGGCAACGggggtttcACT

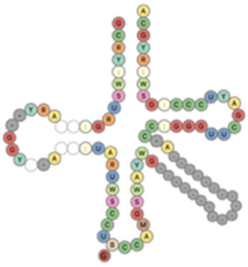

Gossypium_raimondii_Graim2_tRNA-Gly-GCC-5-1 Plant (cotton) (species)

GCGGAA**AT**AGCTTA**ATGGTA**GAGCATAGCCT**TGCCA**AGGCTGAGGtTGAGGG**TTCAAG**TCCCTCCTTCCGCT

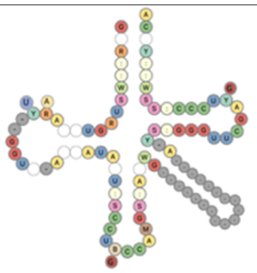

Halalkalicoccus_jeotgali_B3_Hala_jeot_B3_tRNA-Gly-GCC-1-1 Halobacteriaceae archea (species)

GCGCTGGTAGTGTAGTGGTATCACGTGACCTTGCCATGGTCACAaCCTGGGTTCAAATCCCAGCCAGCGCA

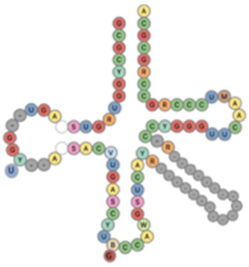

Halanaerobium_hydrogeniformans_Hala_hydr_tRNA-Gly-GCC-1-2 Halanaerobiaceae bacteria (species)

GCGGGAGTAGCTCAGTGGTAGAGCATCACGTTGCCAACGTGAGGGtCGCGAGTTCAAATCTCGTTTCCCGCTCCA

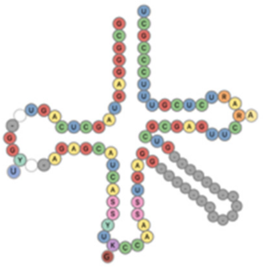

Haloarcula_hispanica_ATCC_33960_CGMCC_12049_tRNA-Gly-GCC-1-1 Halobacteriales archaea (species)

GCGCTGGTAGTGTAGTTGGTATCACGTGACCTTGCCATGGTCACAaCCTGGGTTCAAATCCCAGCCAGCGCA

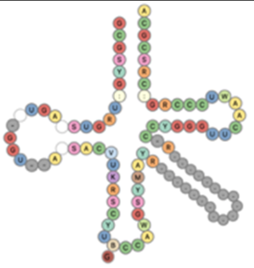

Halobacterium_salinarum_R1_DSM_671_Halo_sali_R1_DSM_671_tRNA-Gly-GCC-1-1 Halobacteriales archaea (species)

GCGCTGGTAGTGTAGTGGTATCACGTGACCTTGCCATGGTCACAaCCTGGGTTCAAA
TCCCAGCCAGCGCA

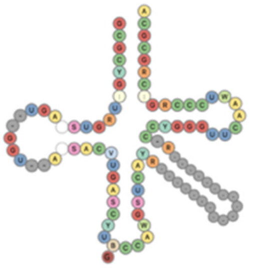

Halodesulfovibrio_sp_MK-HDV_Halo_MK_HDV_tRNA-Gly-GCC-1-1 Proteobacteria

GCGGGAATAACTCAGTGGTAGAGTACAACCTTGCCAAGGTTGGAGtCGCGAGTTCAA
ATCTCGTTTCCCGCTCCA

Haloferax_mediterranei_ATCC_33500_CGMCC_12087_Halo_medi_ATCC_33500_CGMCC_1_2087_tRNA-Gly-GCC-2-1 Euryarchaeota (species)

GCGCTGGTAGTGTAGTGGTATCACGTGACCTTGCCATGGTCACAaCCTGGGTTCAAA
TCCCAGCCAGCGCA

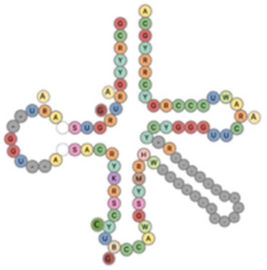

Halogeometricum_borinquense_DSM_11551_PR_3_Halo_bori_DSM_11551_PR_3_tRNA-Gly-GCC-1-2 Euryarchaeota (species)

GCGCTGGTAGTGTAGTGGTATCACGTGACCTTGCCATGGTCACAaCCTGGGTTCAAA
TCCCAGCCAGCGCA

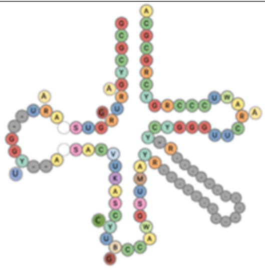

Halomicrobium_mukohataei_DSM_12286_Halo_muko_DSM_12286_tRNA-Gly-GCC-1-1 Halomicrobium (genus)

GCGGTGG**T**AGTGT**A**G**TGGTA**TCACAGGACC**CTGCCA**CGGTCCTAaCGGGGG**TTCAA**
ATCCCCCCCACCGCA

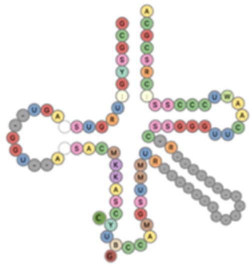

Halomonas_sp_KO116_Halo_KO116_tRNA-Gly-GCC-2-1 Proteobacteria (genus)

GCGGGA**AT**AGCTC**A**G**TGGTA**GAGCATCGCCT**TGCCA**AGGCGAGGGtCGGGAG**TTCA**
AATCTCCTTTCCCGCTCCA

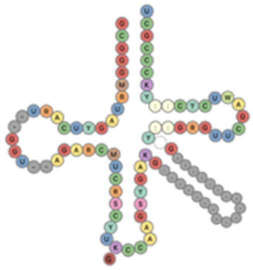

Haloquadratum_walsbyi_C23_Halo_wals_C23_tRNA-Gly-GCC-1-1 Euryarchaeota (species)

GCGCTGG**T**AGTGT**A**G**TGGTA**TCACGTGACCT**TGCCA**TGGTCACAaCCCGGG**TTCAA**A
TCCCGGCCAGCGCA

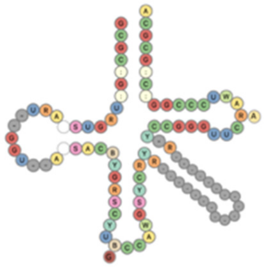

Halorhabdus_tiamatea_SARL4B_Halo_tiam_SARL4B_tRNA-Gly-GCC-1-1 Euryarchaeota (species)

GCGACGGTGGTGTAGTGGTATCACAGGACCCTGCCACGGTCCTAaCCCGAGTTCAA ATCTCGGCCGTCGCA

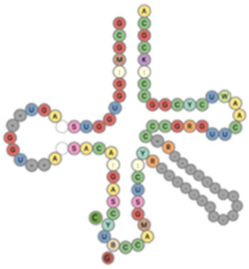

Halorubrum_lacusprofundi_ATCC_49239_Halo_lacu_ATCC_49239_tRNA-Gly-GCC-1-2 Euryarchaeota (species)

GCACCGGTAGTGTAGTGGTATCACGCAACCTTGCCATGGTTGCAaCCCGAGTTCAAA TCTCGGCCGGTGCA

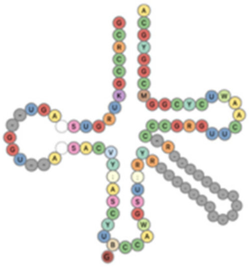

Halostagnicola_larsenii_XH-48_Halo_lars_XH_48_tRNA-Gly-GCC-1-1 Euryarchaeota (species)

GCGTTGGTAGTGTAGTGGTATCACGTGACCTTGCCATGGTCACAaCCTGGGTTCAAA TCCCAGCCAACGCA

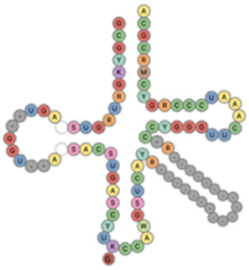

Haloterrigena_turkmenica_DSM_5511_Halo_turk_DSM_5511_tRNA-Gly-GCC-1-2 Euryarchaeota (species)

GCGCTGGTAGTGTAGTGGTATCACGTGACCTTGCCATGGTCACAaCCGGGGTTCAAA TCCCCGCCAGCGCA

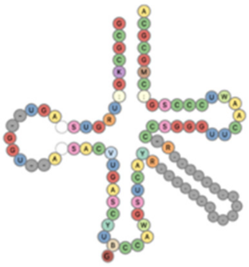

Halovivax_ruber_XH-70_Halo_rube_XH_70_tRNA-Gly-GCC-1-1 Euryarchaeota

GCGCTGG**T**AGTGTAG**TGGTA**TCACGTGACCT**TGCCA**TGGTCACAaCCTGGG**TTCAA**A
TCCCAGCCAGCGCA

Heterocephalus_glaber_Hglab2_tRNA-Gly-GCC-3-1 Mammals (rat) (species)

GCATTGG**TG**GTTC**AGTGGTA**GAATTCTCGC**CTGCCA**CGAGGGAGGCCCGGG**TTCAA**
TTCCCAGCCAATGCA

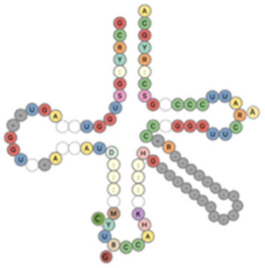

Hyperthermus_butylicus_DSM_5456_Hype_buty_DSM_5456_tRNA-Gly-GCC-1-1 Crenarchaeota (species)

GCGGCGG**T**CGTCTAGCC**TGG**ActAGGACGCCGGC**CTGCCA**AGCCGGCGAtCCCGGG
TTCAAATCCCGGCCGCCGCACCA

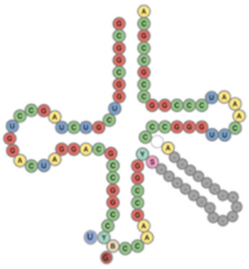

Ignicoccus_hospitalis_KIN4I_Igni_hosp_KIN4_I_tRNA-Gly-GCC-1-1 Crenarchaeota (species)

GCGGCGG**T**CGTCTAGCC**TGG**ActAGGACGCCGGC**CTGCCA**CGCCGGAGAtCCCGGG
TTCAAATCCCGGCCGCCGCACCA

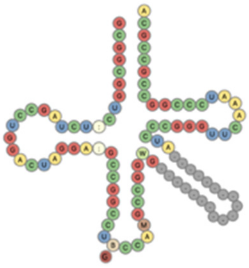

Ignisphaera_aggregans_DSM_17230_Igni_aggr_DSM_17230_tRNA-Gly-GCC-1-1 Crenarchaeota

GCGGCGG**T**CGTCTAGCC**TGGT**ctAGGACGCCGGC**CTGCCA**AGCCGGAGAtCCCGGG**T**
TCAAATCCCGGCCGCCGCACCA

Isosphaera_pallida_ATCC_43644_Isos_pall_ATCC_43644_tRNA-Gly-GCC-1-1 Planctomycetota bacteria (species)

GCGGGAG**T**AGCTC**A**GG**GGTA**GAGCGCCACGT**TGCCA**ACGTGGTTGtCGTGGG**TTCA**
AATCCCATCTCCCGCT

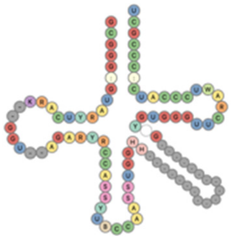

Laccaria_bicolor_S238N-H82_Lacc_bico_S238N_H82_tRNA-Gly-GCC-4-1 Fungus (species)

GCATTA**ATG**GGGT**A**G**TGGTA**ACCTGGGTCAT**TGCCA**tgaggatttcatatctgtttgtgtttacctataT
TGACCTGcTGGGGG**TTCAA**TTCCCTCTTAATGCA

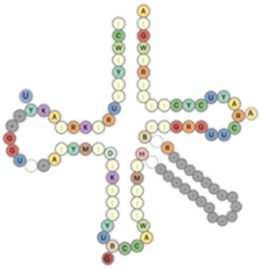

Lacinutrix_sp_5H-3-7-4_Laci_5H_3_7_4_tRNA-Gly-GCC-1-1 Flavobacteriaceae

GCGAAAG**T**AGCTC**A**GG**GGTA**GAGCATCACCT**TGCCA**AGGTGGAGGtCGTGGG**TTCA**
AATCCCATCTTTCGCT

Lactobacillus_helveticus_DPC_4571_Lact_helv_DPC_4571_tRNA-Gly-GCC-3-1 Firmicutes bacteria (species)

GCGGAAG**T**AGTTC**A**G**TGGTA**GAACATCACCT**TGCCA**TGGTGGGGGtCGCGGG**TTCAA**
ATCCCGTCTTCCGCT

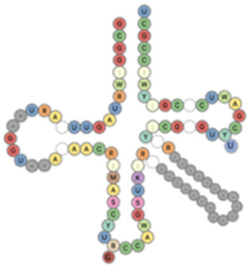

Latimeria_chalumnae (cœlacanthe)_Lchal1_tRNA-Gly-GCC-3-1 Fish (species)

GCATTGG**TG**GTTC**A**G**TGGTA**GAATTCTCGC**CTGCCA**CGCGGGAGaCCCGGG**TTCAA**
TTCCCGGCCAATGCA

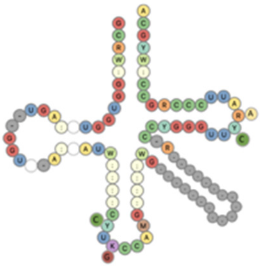

Lawsonia_intracellularis_N343_Laws_intr_N343_tRNA-Gly-GCC-1-1
Plant (magnoliophyta) (species)

GCGGGAG**T**AACTC**A**G**TGGTA**GAGTGCAACCT**TGCCA**AGGTTGAAGtCGCGGG**TTCAA**
ATCCCGTCTCCCGCTCCA

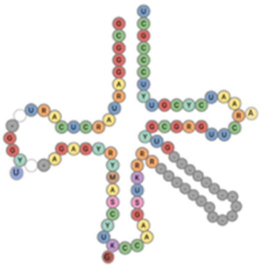

Leptospira_biflexa_serovar_Patoc_strain_Patoc_1_Ames_tRNA-Gly-GCC-1-1 Spirochaetae bacteria (species)

GCGGGA**AT**AGCTC**A**GC**GGTA**GAGCATCTCCT**TGCCA**AGGAGAGGGtCGCGGG**TTCA**
AGTCCCGTTTCCCGCT

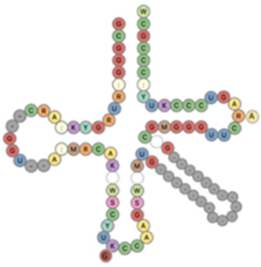

Loxodonta_africana_Lafri3_tRNA-Gly-GCC-6-1 Mammals (elephant) (species)

GCGTTGG**TG**GTCC**A**G**TGGTA**GAATTCTTGC**CTGCCA**TGCAGGAGGCCCAGG**TTCAA**
TTCCTGGCCAATGCA

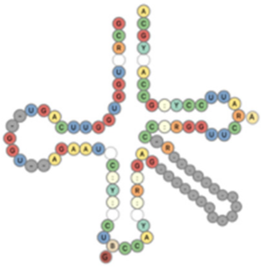

Macaca_mulatta_Mmula8_tRNA-Gly-GCC-11-1 Mammals (primates) (species)

GCATGGG**TG**GTTCAG**TGGTA**GAATTCTTGC**CTGCCA**CGCGGGAGGCCCGGG**TTCAA**
TTCCTGGCCCATACA

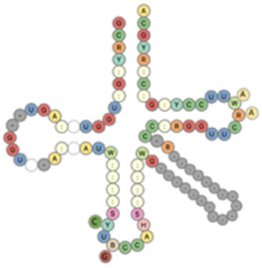

Macropus_eugenii_Meuge2_tRNA-Gly-GCC-9-1 Macropodinae (kangaroo)

GGTTaccgc**ATG**GATGGTTCAG**TGGTA**GAATCCTTCC**CTGCCA**CGCGGGAGGTCCAGG
TTCAATTCCTGGCTCGACCA

Medicago_truncatula_A17_Mtrun4_tRNA-Gly-GCC-2-1 Plant (tracheophytes) (species)

GCACCAG**TG**GTCT**AGTGGTA**GAATAGTACC**CTGCCA**TGGTACAGaCCCGGG**TTCAA**T
TCCTGGCTGGTGCA

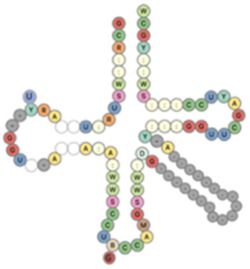

Melopsittacus_undulatus_Mundu1_tRNA-Gly-GCC-2-2 Aves (parakeet) (species)

GCATTGG**TG**GTTC**AGTGGTA**GAATTCTCGC**CTGCCA**CGCGGGAGGCCCGGG**TTCAA**
TTCCCGGCCAATGCA

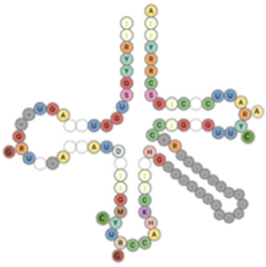

Metallosphaera_sedula_CuR1_Meta_sedu_CuR1_tRNA-Gly-GCC-1-1 Thermoproteota (archaea) (species)

GCGGCCG**T**AGTCTAGCC**TGG**AttAGGACGC**CTGCC**TGCCACGCAGGAGGtCCCGGG**T**
TCAAATCCCGGCGGTCGCA

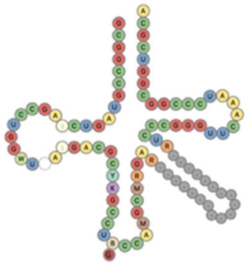

Methanobacterium_formicicum_BRM9_Meth_form_BRM9_tRNA-Gly-GCC-1-1 Euryarchaeota (species)

GCGGCGT**T**AGTCCAGCC**TGGT**TAAGACACTGGC**CTGCCA**CGCCAGCGACCCGGG**TT**
CAAATCCCGGACGCCGCA

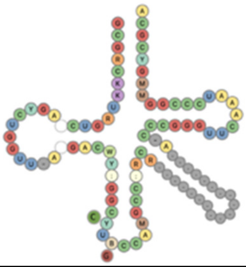

Methanobrevibacter_ruminantium_M1_Meth_rumi_M1_tRNA-Gly-GCC-1-1 Euryarchaeota (species)

GCGGTGTTAGTCCAGCCTGGTtAAGACTCTAGCCTGCCACGTTAGAGACCCGGGTTCAAATCCCGGACGCCGCA

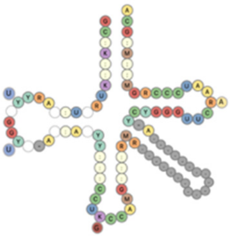

Methanocaldococcus_fervens_AG86_Meth_ferv_AG86_tRNA-Gly-GCC-1-1 Euryarchaeota (species)

GCGGCCTTGGTGTAGCCTGGTAACACACGGGCCTGCCACGCCCGGAcCCCGGGTTCAAATCCCGGAGGCCGCACCA

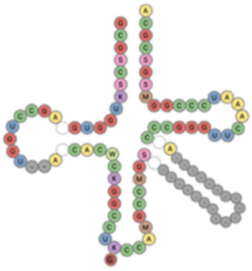

Methanocella_arvoryzae_MRE50_Meth_arvo_MRE50_tRNA-Gly-GCC-1-1 Methanocellales archaea (species)

GCGCCGGTAGTGTAGTGGTtATCACTGTAGCTTGCCAAGCTATAGACTCGGGTTCAATTCCCGACCGGCGCACCA

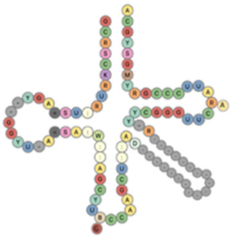

Methanococcus_maripaludis_C5_Meth_mari_C5_tRNA-Gly-GCC-1-1 Methanococcaceae archaea (species)

GCGGCTTTGATGTAGACTGGTATCATACGGCCCTGCCACGGCCGACACCCGGGTTCAAATCCCGGAGGCCGCA

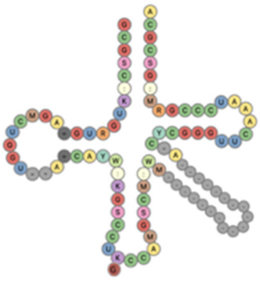

Methanococcus_voltae_A3_Meth_volt_A3_tRNA-Gly-GCC-1-1 Methanococcaceae archaea (species)
GCGGCCTTGATGTAGTGGTATCATACGGCCCTGCCACGGCCGATACCCGGGTTCAAATCCCGGAGGCCGCA

Methanopyrus_kandleri_AV19_Meth_kand_AV19_tRNA-Gly-GCC-1-1 Euryarchaeota

GCGGCCGCAGTCTAGTC**TGGTA**GGACGCGGGC**CTGCC**GAGCCCGTGGCCCGGG**TT**
CAAATCCCGGCGGCCGCACCA

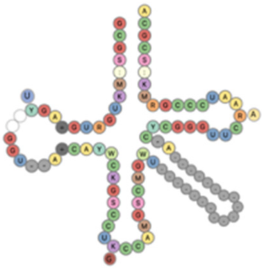

Methanosarcina_lacustris_Z-7289_Meth_lacu_Z_7289_tRNA-Gly-GCC-1-1
Methanosarcinales archaea (species)

ACATCAG**T**AGTGT**A**GC**GGT**cATCACCGGGCGT**TGCCA**ACGCTCGAaCTCGGG**TTCAA**
ATCCCGACTGGTGTA

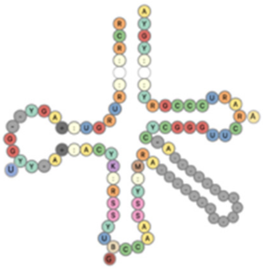

Methanosphaera_stadtmanae_DSM_3091_Meth_stad_DSM_3091_tRNA-Gly-GCC-1-1
Methanobacteriaceae archaea (species)

GCAGCG**AT**AGTCCAGGC**TGG**CtAAGACTCTACC**CTGCCA**CGGTAGTGACCCGGG**TTC**
AAATCCCGGTCGTTGCA

Methanothermobacter_sp_CaT2_Meth_CaT2_tRNA-Gly-GCC-1-1
Methanobacteriaceae archaea (genus)

GCGGCGT**T**AGTCCAGCC**TGGT**TAAGACACTGGC**CTGCCA**CGCCAGCGACCCGGG**TT**
CAAATCCCGGACGCCGCA

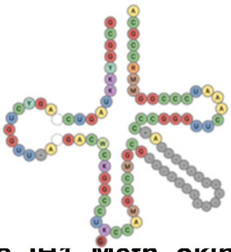

Methanothermococcus_okinawensis_IH1_Meth_okin_IH1_tRNA-Gly-GCC-1-1
Methanocaldococcaceae archaea (species)

GCGGCCT**TG**ATGTAGCC**TGGTA**TCATACGGCC**CTGCCA**CGGCCGATACCCGGG**TTC**
AAATCCCGGAGGCCGCA

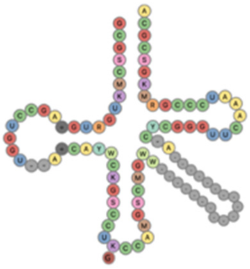

Methanothermus_fervidus_DSM_2088_Meth_ferv_DSM_2088_tRNA-Gly-GCC-1-1 Methanothermaceae archaea

GCGGCGTTAGTCCAGCCTGGTtAAGACGCTGGCCTGCCACGCCAGTGACCCGGGTTCAAATCCCGGACGCCGCA

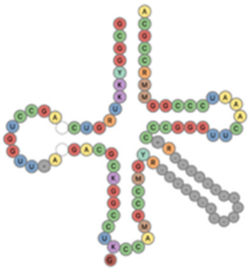

Methanotorris_igneus_Kol_5_Meth_igne_Kol_5_tRNA-Gly-GCC-1-1 Methanocaldococcaceae (archaea) (species)

GCGGCCTTGATGTAGCCTGGTAACATACGGGCCTGCCACGCCCGTTcCCCGGGTTCAAATCCCGGAGGCCGCACCA

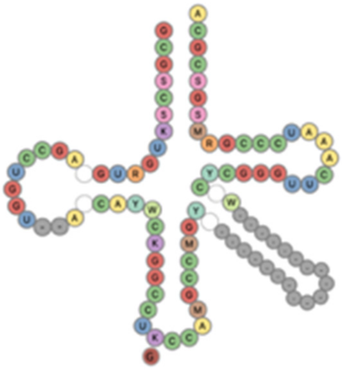

Methylacidiphilum_infernorum_V4_Meth_infe_V4_tRNA-Gly-GCC-1-1 Methylacidiphilales archaea

GCGGGTGTAGCTCAGTTGGTAGAGCGCAACCTTGCCAAGGTTGACGtCGCGGGTTCAAATCCCGTCACCCGCT

Microcoleus_sp_PCC_7113_Micr_PCC_7113_tRNA-Gly-GCC-2-1 Cyanobacteria

GCGGGTGTGATGTAGTGGCtAGCATCTGAGTCTGCCAGTCTCAGCgCATGGGTTCAAGTCCCATCATCCGCT

Monodelphis_domestica_Mdome5.1_tRNA-Gly-GCC-3-1 Mammals (dolphin) (species)

GCATTGGTGGTTCAGTGGTAGAATTCTCGCCTGCCACGCGGGGGCCTGGGTTCAATTCCCGGCCAATGCA

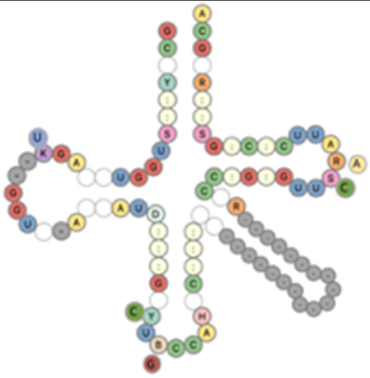

Moorella_thermoacetica_ATCC_39073_Moor_ther_ATCC_39073_tRNA-Gly-GCC-1-1 Bacillota (species)

GCGGAAG**T**AGCTC**A**G**TGGTA**GAGCATCGCCT**TGCCA**AGGCGAGGGcCGCGGG**TTCA**
AATCCCGTCTTCCGCTCCA

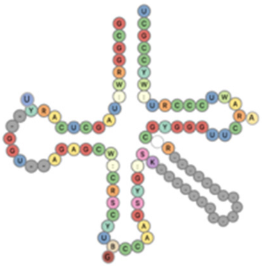

Mrakia_frigida_Nwmf-AP1_Mrak_frig_Nwmf_AP1_tRNA-Gly-GCC-1-1 Fungus (species)

ATAGGTA**TG**GTGA**A**G**TGG**CtATCATTCGCGCT**TGCCA**TGCGTGAGGCAGGGG**TTCAA**
TTCCCCTTACCTATA

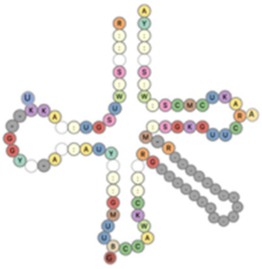

Mus_caroli_CAROLIEiJ_MusCAROLI_EiJ_1509_tRNA-Gly-GCC-34-1 Mammals (mouse)

GCATAAG**TG**GTTC**A**G**TGGTA**GAATTCTCAC**CTGCCA**TTTGGGAGGCCCAGG**TTCAA**T
TCCAGGCCCATtGCA

Mus_musculus_AJ_MusA_J_1509_tRNA-Gly-GCC-2-1 Mammals (mouse) (species)

GCATGGG**TG**GTTC**A**G**TGGTA**GAATTCTCAC**CTGCCA**TGAGGGAGGCCCAGG**TTCAA**
TTCCAGGCCCATtGCA

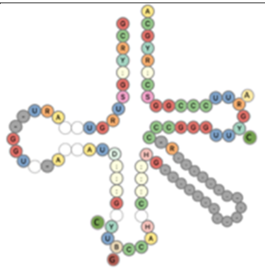

Mus_pahari_PAHARIEiJ_MusPAHARI_EIJ_1509_tRNA-Gly-GCC-392-1 Mammals (mouse)

GAGATGGCTCAGTGGTtaAGGATACTGACTGCCATTCCAGATGaCCTGGGTTCAATTCCTAGCAACTGCA

Mus_spretus_SPRETEiJ_MusSPRET_EiJ_1509_tRNA-Gly-GCC-4-1 Mammals (mouse)

GCATGGGTGGTTCAGTGGTAGAATTCTCACCTGCCATGAGGGAGGCCCAGGTTCAATTCCAGGCCCATtGCA

Mycoplasma_agalactiae_5632_Myco_agal_5632_tRNA-Gly-GCC-1-1 Tenericutes bacteria (species)

GCAAATGTAGTTCAATGGTAGAACACCAGCTTGCCATGCTGGATaCGGGGGTTCAATTCCCCTCATTTGCTCCA

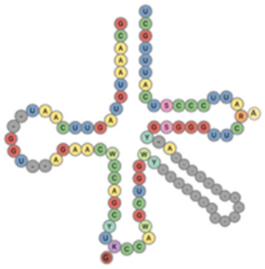

Myxococcus_stipitatus_DSM_14675_Myxo_stip_DSM_14675_tRNA-Gly-GCC-1-1 Deltaproteobacteria (species)

GCGGGAATAGCTCAGCGGTAGAGCATCGCCTTGCCAAGGCGAGGGtCGAGGGTTCAAATCCCTTTTCCCGCTCCA

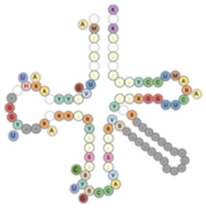

Naegleria_gruberi_NEG-M_Naeg_grub_NEG_M_tRNA-Gly-GCC-1-6 Eukaryota (amoeba)

GCGTCGATGGTTTAGTGGTAGAATACGGCGTTGCCATCGCCGTGaCCCGGGTTCAATTCCCGGTCGACGCA

Natrialba_magadii_ATCC_43099_Natr_maga_ATCC_43099_tRNA-Gly-GCC-1-1 Halobacteriaceae archaea (species)

GCGCTGGTAGTGTAGTGGTATCACGTGACCTTGCCATGGTCACAaCCTGGGTTCAAATCCCAGCCAGCGCA

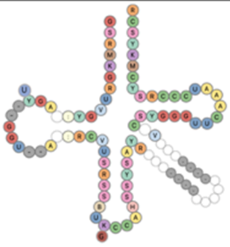

Natrinema_pellirubrum_DSM_15624_Natr_pell_DSM_15624_tRNA-Gly-GCC-1-1 Halobacteriaceae archaea (species)

GCGCTGG**T**AGTGT**A**G**TGGTA**TCACGTGACCT**TGCCA**TGGTCACAaCCTGGG**TTCAA**A
TCCCAGCCAGCGCA

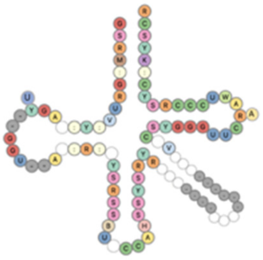

Natronococcus_occultus_SP4_Natr_occu_SP4_tRNA-Gly-GCC-1-1 Halobacteriaceae archaea (species)

GCGCTGG**T**AGTGT**A**G**TGGTA**TCACGTGACCT**TGCCA**TGGTCACAaCCTGGG**TTCAA**A
TCCCAGCCAGCGCA

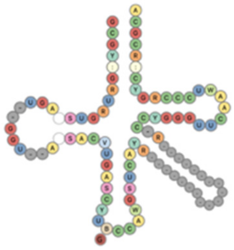

Natronomonas_moolapensis_8811_Natr_mool_8_8_11_tRNA-Gly-GCC-1-1 Halobacteriaceae archaea (species)

GCGCTGG**T**AGTGT**A**G**TGGTA**TCACGTGACCT**TGCCA**TGGTCACAaCCGGGG**TTCAA**A
TCCCCGCCAGCGCA

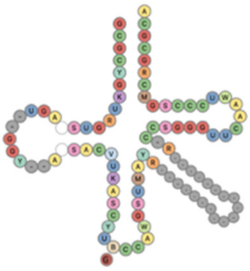

Nicotiana_tabacum_K326_Ntaba_K326_tRNA-Gly-GCC-4-1 Plant (solanacea)

GCGGAA**AT**AGCTT**AATGGTA**GAGCATAGCCT**TGCCA**AGTCTAAGAtTGAGGG**TTCAA**
GTCCCTCTTTCCGCT

Nitrososphaera_viennensis_EN76_Nitr_vien_EN76_tRNA-Gly-GCC-1-1 Nitrosphaeraceae archaea (species)

GCGACGG**T**CGTCCAGTTT**TGGT**ctAGGACATCAGAT**TGCCA**ATCTGGTAaCCCGGG**TTC**
AAATCCCGGCCGTCGCACCA

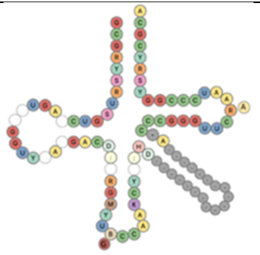

Nonlabens_dokdonensis_DSW-6_Nonl_dokd_DSW_6_tRNA-Gly-GCC-1-1 Flavobacteria (species)

GCGAAAGTAGCTCAGGGGTAGAGCATCACCTTGCCAAGGTGGAGGtCGCGAGTTCA
AATCTCGTCTTTCGCT

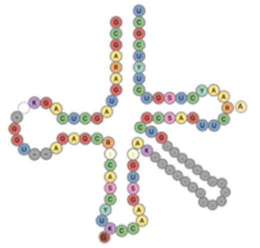

Oreochromis_niloticus_Onilo2_tRNA-Gly-GCC-11-1 Fish (species)

CACTTCATAGTACAACGGTTCAGACATTTGCTTGCCATGCAAAAGGtCACTGGTTCAA
TTTTAGCTGGAGattaaA

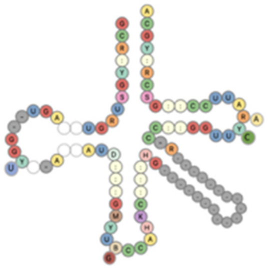

Orientia_tsutsugamushi_str_Boryong_Orie_tsut_Boryong_tRNA-Gly-GCC-1-1 Rickettsiaceae bacteria (species)

GCGGATGTAGCTCAGTGGTAGAGCGTTACTTTGCCAAGGTAAAAGtCGTGGGTTCAA
TTCCCATCATCCGCT

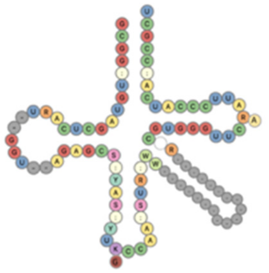

Oryctolagus_cuniculus_Ocuni2_tRNA-Gly-GCC-21-1 Mammals (rabbit) (species)

GGAGCTGTGGCATAGTGGGTAAAGCCGCTGCCTGCCATGCCAGCATCCCATATGGg
CGCAGGTTCAAGTCCCGGCTGCTCCA

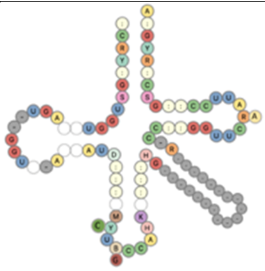

Oryzias_latipes_Olati3_tRNA-Gly-GCC-2-1 Fish (species)

GCATTGG**TG**GTAT**A**G**TGGTA**GCATAGCTGCT**TGCCA**TGCAGTTGaCCTGGG**TTCAA**T
TCCCAGCCAATGCA

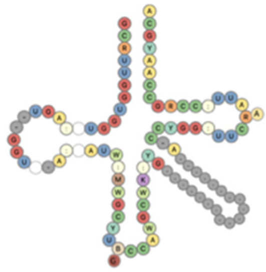

Oscillatoria_acuminata_PCC_6304_Osci_acum_PCC_6304_tRNA-Gly-GCC-2-1 Cyanobacteria (species)

GCGGGTG**TG**ATGT**A**G**TGGTA**AGCATCTGAGT**CTGCCA**GTCTCAGTGCATGGG**TTCAA**
ATCCCATCATCCGCT

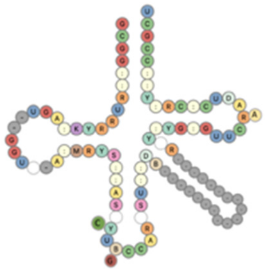

Otolemur_garnettii_Ogarn3_tRNA-Gly-GCC-5-1 Mammals (primates) (species)

GCATGGG**TG**GTTC**A**G**TGGTA**GAATTCTCGC**CTGCCA**CGCGGGAGGCCCGGG**TTCAA**
TTCCCGGCCCATGCA

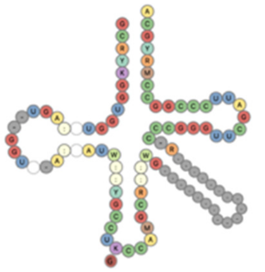

Ovis_aries_Oarie4_tRNA-Gly-GCC-1572-1 Mammals (sheep) (species)

GCATGAG**TG**GTTC**A**G**TGGTA**GAATTCTCAC**CTGCCA**CGTGGGAAgCCCAGA**TTCAA**T
TCCCAGTCCATGCA

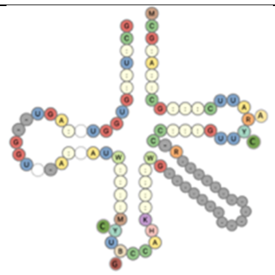

Palaeococcus_pacificus_DY20341_Pala_paci_DY20341_tRNA-Gly-GCC-1-1 Thermococcaceae archaea (species)

GCGGTGG**T**AGTCTAGCC**TGG**CctAGGACTGCGGC**CTGCCA**CGCCGCAAgCCCGGG**TT**
CAAATCCCGGCCACCGCACCA

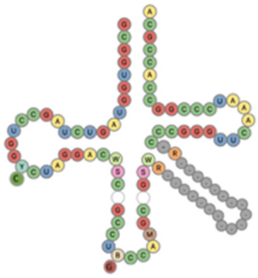

Parachlamydia_acanthamoebae_UV-7_Para_acan_UV_7_tRNA-Gly-GCC-2-1 Chlamydiae bacteria (species)

GCGGGTG**T**AGCTC**A**G**TGGTA**GAGCATCACGT**TGCCA**ACGTGAGGGtCGTGAG**TTCAA**
GCCTCATCACCCGCT

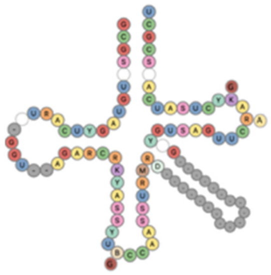

Paracoccidioides_brasiliensis_Pb03_Para_bras_Pb03_tRNA-Gly-GCC-2-1 Fungus (species)

GCATCAT**TG**GTCT**A**G**TGGTA**GAATTCATCGT**TGCCA**tccaccgctgt**TTCAA**acggcgttcggaT
TCGATGAGGCC

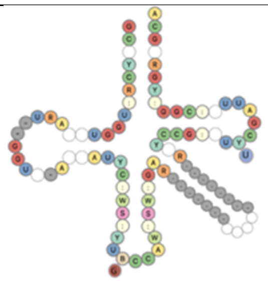

Petromyzon_marinus_Pmari2_tRNA-Gly-GCC-4-2 Agnatha (lamprey)

GCATCGG**TG**GTTC**A**G**TGGTA**GAATTCTCGC**CTGCCA**CGCGGGAGGCCCGGG**TTCAA**
TTCCCGGCCGATGCA

Pleurotus_ostreatus_PC15_Pleu_ostr_PC15_tRNA-Gly-GCC-3-2 Fungus (species)

GCATCA**ATG**GGGT**A**G**TGGTA**ACCTGGGTCGT**TGCCA**ttgagtataTCGACCCGcCGCGAG
TTCAATTCTCGCTTGATGCA

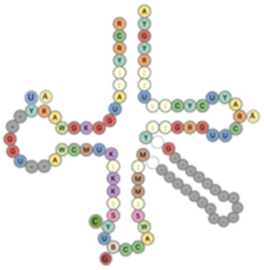

Pleurozium_schreberi_Blido_Pschr1_tRNA-Gly-GCC-2-1 Plant (moss)

GCACCAG**TG**GTCT**A**G**TGGTA**GAATAGTACC**CTGCCA**CGGTACAGaCCCGGG**TTCAA**T
TCCCGGCTGGTGCA

Plicaturopsis_crispa_FD-325_SS-3_Plic_cris_FD_325_SS_3_tRNA-Gly-GCC-2-1 Fungus (species)

GCGTTA**ATG**GGGT**A**G**TGGTA**ACCTTGGTCGT**TGCCA**ttagcacaTTCGACCAGcCGGGG
G**TTCAA**TTCCCCCTTAATGCA

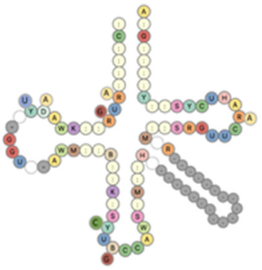

Pohlia_nutans_NOL_Pnuta1_tRNA-Gly-GCC-16-1 Plant (moss)

GAATTGGg**T**ttTgaGGTCT**A**G**TGGTA**GAATAGTATC**CTGCCA**CGGTACAGaCCCGGG**TT**
CAATTCCCGGCCGGTGCA

Polaribacter_sp_MED152_Pola_MED152_tRNA-Gly-GCC-1-1 Flavobacteria

GCGAAAGTAGCTCAGGGGTAGAGCATCACCTTGCCAAGGTGAGGGtCGCGGGTTCAAATCCCGTCTTTCGCT

Pristionchus_pacificus_Ppaci4_tRNA-Gly-GCC-2-1 Nematodes (species)

TCTCTTGTAGTATAGTGGTtAGTATCCGCGCCTGCCACGTGCGAGaCCCGGGTTCAATTTCCGGCCAGAGAG

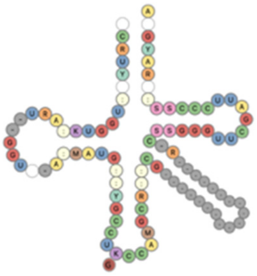

Procavia_capensis_Pcape2_tRNA-Gly-GCC-2068-1 Mammals (daman) (species)

GGGGCCTcgtTGGCTTAGTGGTTAAGTACTTGATTGCCAACCAAGAGGtTGGTGGTTCAAACCCAGCAGCTCCA

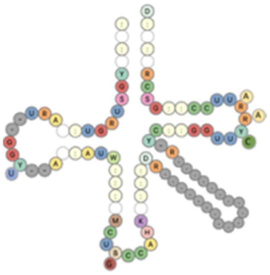

Psychroflexus_torquis_ATCC_700755_Psyc_torq_ATCC_700755_tRNA-Gly-GCC-1-1 Flavobacteria (species)

GCGAAAGTAGCTCAGCGGTAGAGCACCACCTTGCCAAGGTGGGGGtCGCGGGTTCAAATCCCGTCTTTCGCT

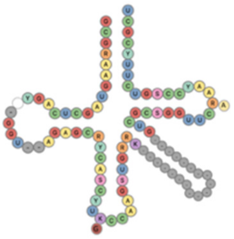

Puccinia_striiformis_f_sp_tritici_CY32_Pucc_stri_f_tritici_CY32_tRNA-Gly-GCC-8-1 Fungus

GCaTCTGGTGTCAGTTGGGACAGCATCAGACTGCCAacatgggctttcactcatgcATCTGAAGGtCCAGTGTTCAAGCCACTGTTGGGACA

Pyrenochaeta_lycopersici_CRA-PAV_ER_1211_Pyre_lyco_CRA_PAV_ER_1211_tRNA-Gly-GCC-2-1 Fungus

GGCACTTTGGCGGAGTGGTtAACGCGTATGTCTGCCAactaccaccaTTCAActatcttgctaactcagtc

Pyrobaculum_aerophilum_str_IM2_Pyro_aero_IM2_tRNA-Gly-GCC-1-1 Thermoproteaceae archaea

GCGGCGG**T**AGTCTAGCC**TGGT**ttAGGATGGCGGC**CTGCCA**AGCCGTTGAtCCCGGG**T**
TCAAATCCCGGCCGCCGCACCA

Pyrococcus_abyssi_GE5_Pyro_abys_GE5_tRNA-Gly-GCC-1-1 Euryarchaoeta archaea (species)

GCGGTGG**T**AGTCTAGCC**TGG**CctAGGACGCCACC**CTGCCA**AGGTGGAGACCCGGG**T**
TCAAATCCCGGCCACCGCACCA

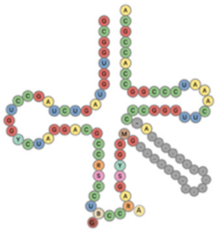

Pyrolobus_fumarii_1A_Pyro_fuma_1A_tRNA-Gly-GCC-1-1 Crenarchaeota archea (species)

GCGGCGG**T**CGTCTAGCC**TGG**ActAGGACGCCGGC**CTGCCA**AGCCGGAGAtCCCGGG
TTCAAATCCCGGCCGCCGCACCA

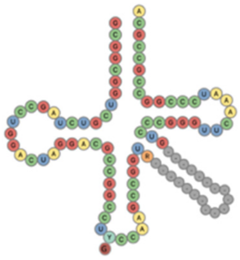

Rattus_norvegicus_Rnorv6_tRNA-Gly-GCC-1278-1 Mammals (rat) (species)

GaGAg**ATG**GCTC**AATGGT**taAGAGCACTGA**CTGCC**GtgctcTTCCAGAGGcCGTGAG**TTC**
AAGTCTCAGTAACCA

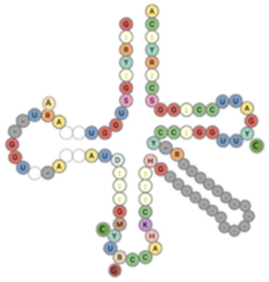

Rhizobium_gallicum_bv_gallicum_R602_Rhiz_gall_bv_gallicum_R602_tRNA-Gly-GCC-1-1 Alphaproteobacteria (genus)

GCGGGTG**T**AGCTC**A**GG**GGTA**GAGCACAACCT**TGCCA**AGGTTGGGGtCGAGGG**TTCA**
AATCCCTTCGCCCGCTCCA

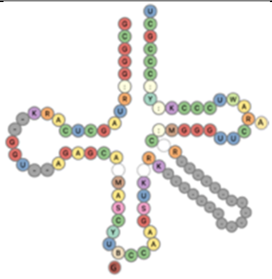

Rhopilema_esculentum_Rescu1_tRNA-Gly-GCC-8-1 Cnidaria (jellyfish)

GCCACGGTAGCCGAGTGGTtAAGGTGCTCGACTGCCATGCCAGCACTCTgGGTTCAA
TCCTGGCTCGcaGACA

Rhytidiadelphus_loreus_Rlore1_tRNA-Gly-GCC-2-1 Bryophyta

GCACCAGTGGTCTAGTGGTAGAATAGTACCCTGCCACGGTACAGaCCCGGGTTCAAT
TCCCGGCTGGTGCA

Rickettsia_massiliae_MTU5_Rick_mass_MTU5_tRNA-Gly-GCC-1-1 Rickettsia bacteria

GCGGGTGTAGCTCAGGGGTAGAGCGCTACCTTGCCAAGGTCGAAGtCGAGGGTTCA
AATCCCTTCACCCGCTCCA

Rothia_dentocariosa_ATCC_17931_Roth_dent_ATCC_17931_tRNA-Gly-GCC-1-1 Actinobacteria (species)

GCGGTTGTAGCTCAGTTGGTAGAGCACCACCTTGCCAAGGTGGATGtCGCGAGTTCA
AGTCTCGTCAACCGCT

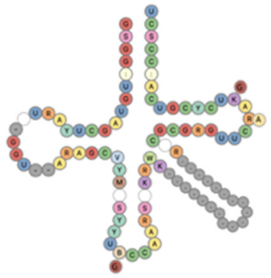

Ruminiclostridium_thermocellum_ATCC_27405_Rumi_ther_ATCC_27405_tRNA-Gly-GCC-2-1 Bacillota

GCGGGTTTAACTCAGTGGTAGAGTGTCACCTTGCCAAGGTGAAAGtCGCGAGTTCAA
ATCTCGTAACCCGCTCCA

Saccharolobus_shibatae_B12_Sacc_shib_B12_tRNA-Gly-GCC-1-1 Thermoproteota archaea

GCGGCCGTAGTCTAGCCTGGAttAGGACGCCTGCCTGCCACGCAGGAGGtCCCGGGT
TCAAATCCCGGCGGCCGCA

Salinarchaeum_sp_Harcht-Bsk1_Sali_Harcht_Bsk1_tRNA-Gly-GCC-1-1 Euryarchaeota
GCGTCGGTAGTGTAGTGGTATCACGTGACCCTGCCACGGTCGCAaCCCGAGTTCAA
ATCTCGGCCGACGCA

Schizosaccharomyces_octosporus_yFS286_Schi_octo_yFS286_tRNA-Gly-GCC-2-1 Fungus (species)

GCTTTGG**TG**GTTT**A**G**TGGTA**TAATGCTTCGT**TGCCA**TCGAAGCGaCCCGGG**TTCAA**T
TCCCGGCCGAAGCA

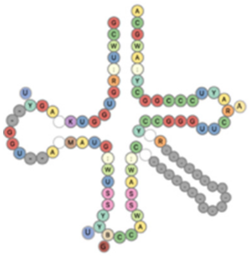

Sclerotinia_sclerotiorum_1980_UF-70_Scle_scle_1980_UF_70_tRNA-Gly-GCC-7-1 Fungus (species)

GCGTTTG**TG**GTTTA**ATGGTA**AAATCCATCGT**TGCCA**tcagggtctcttgttctcatg**TTCAA**tagata
atgaataatgTCG

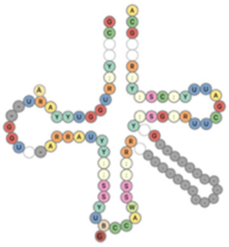

Serratia_marcescens_SM39_Serr_marc_SM39_tRNA-Gly-GCC-3-1 Enterobacteria (species)

GCGGGA**AT**AGCTCAGT**TGGTA**GAGCGCAACCT**TGCCA**AGGTTGAGGtCGCGAG**TTCA**
AGCCTCGTTTCCCGCTCCA

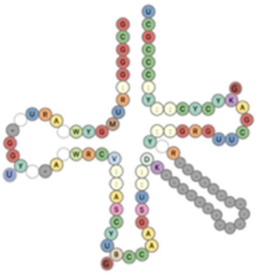

Siansivirga_zeaxanthinifaciens_CC-SAMT-1_Sian_zeax_CC_SAMT_1_tRNA-Gly-GCC-1-1 Flavobacteria (species)

GCGAAAG**T**AGCTCAGG**GGTA**GAGCATCACCT**TGCCA**AGGTGAGGGtCGCGGG**TTCA**
AATCCCGTCTTTCGCT

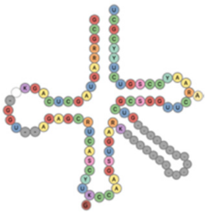

Simkania_negevensis_Z_Simk_nege_Z_tRNA-Gly-GCC-1-1 Chlamydiota bacteria (species)

GCGGGTG**T**AGCTC**A**G**TGGTA**GAGCATCACGT**TGCCA**ACGTGAGGGtCGTGAG**TTCAA**
ATCTCATCACCCGCT

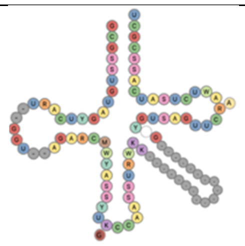

Singulisphaera_acidiphila_DSM_18658_Sing_acid_DSM_18658_tRNA-Gly-GCC-1-1 Planctomycetota bacteria (species)

GCGGGAG**T**AGCTCAGG**GGTA**GAGCGCCACGT**TGCCA**ACGTGGTTGtCGTGGG**TTCA**
AATCCCATCTCCCGCT

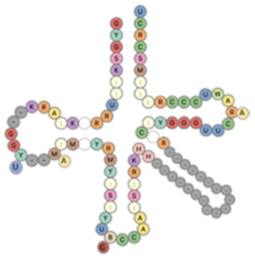

Solanum_lycopersicum_Heinz_1706_Slyco3_1_tRNA-Gly-GCC-1-1 Plant (tomato)

GCGGAA**AT**AGCTT**AATGGTA**GAGCATAGCCT**TGCCA**AGGCTAAGGtTGAGGG**TTCAA**
GTCCCTCCTTCCGCT

Sorangium_cellulosum_So0157-2_Sora_cell_So0157_2_tRNA-Gly-GCC-1-2 Myxococcales bacteria (species)

GCGGGAG**T**AACTC**A**G**TGGTA**GAGTGCAACCT**TGCCA**AGGTTGACGtCGCGGG**TTCAA**
ATCCCGTCTCCCGCTCCA

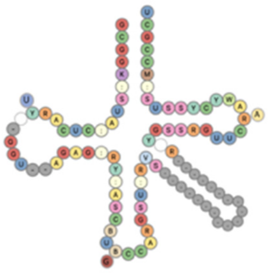

Sorex_araneus_Saran2_tRNA-Gly-GCC-29-1 Mammals (shrew) (species)

GCTcG**AT**TC**A**G**TGGTA**GAATTCTCGG**CTGCCA**CGTGGGAGGCCCGGG**TTCAA**TTCC
CGGcccaTGCA

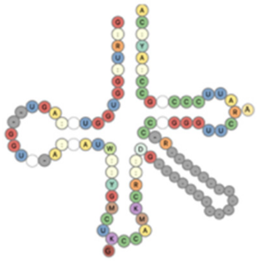

Spermophilus_tridecemlineatus_Strid2_tRNA-Gly-GCC-4615-1 Mammals (squirrel)

GgGTtTTAGCTCAGTGGTAtAGAGCTTGCcGCTTGCCTAGCatatGCGAGGcTGTAGGTTCAATCCCTAgTACAACA

Sphaerobolus_stellatus_SS14_Spha_stel_SS14_tRNA-Gly-GCC-1-1 Fungus (species)

GCACTAATGGGGTAGTGGTAACCTTGGTCGTTGCCAtcgagcacaaTCGACCAGcCAGGGGTTCAATTCCCCTTTAGTGCA

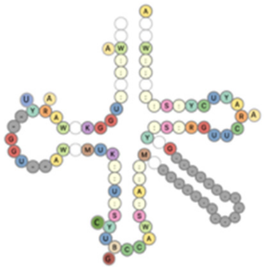

Sphaerochaeta_coccoides_DSM_17374_Spha_cocc_DSM_17374_tRNA-Gly-GCC-1-1 Spirochaetota bacteria (species)

GCGAGAGTAGCTCAGTGGTAGAGCTCCACCTTGCCAAGGTGGATGtCGCGGGTTCAAATCCCGTCTCTCGCT

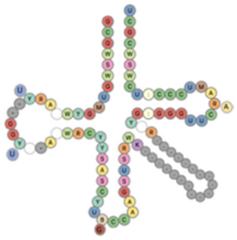

Sphaerulina_populicola_P0202b_p0202b_Spha_popu_P02_02b_p02_02b_tRNA-Gly-GCC-1-1 Fungus (species)

GCACTAGTGGTTTAGTGGTAAAATTGCCGCTTGCCAtccaaattggatcctTTCAAaccagggttctaaag

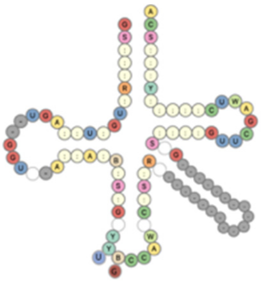

Sphagnum_fallax_MN_Sfall1_tRNA-Gly-GCC-5-1 Bryophyta

GCGGAAATAACTTAATGGTAGAGTATAGCCTTGCCAAGGCTGAGGtTGAGGGTTCAAGTCCCTTTTTTCGCT

Spirochaeta_thermophila_DSM_6192_Spir_ther_DSM_6192_tRNA-Gly-GCC-1-1 Spirochaetota bacteria (species)

GCGGGAGTAGCTCAGTGGTAGAGCTCCACCTTGCCAAGGTGGATGtCGCGGGTTCAAGTCCCGTCTCCCGCT

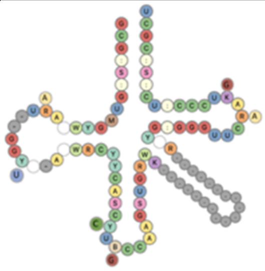

Spirogloea_muscicola_CCAC_0214_Smusc1_tRNA-Gly-GCC-15-1 Algae

GTGGAAGTAGGTTACTGGTAGAGTATAGCATTGCCAAGGCTGAGGtTGAGGGTTCAA
GTCCCTTGTTCCGCT

Spodoptera_frugiperda_Sfrug1_tRNA-Gly-GCC-4-1 Lepidoptera

TGCACaGTTGGCCCAGTGGCTGGGCAACTGACTGCCGTGCAACGTGtCGCGGGTTCA
ATTCTCGCACGCACAA

Stereum_hirsutum_FP-91666_SS1_Ster_hirs_FP_91666_SS1_tRNA-Gly-GCC-2-3 Fungus (species)

GCATCAATGGGGTAGTGGTAACCTGGGTCGTTGCCAtcatgtTTCGAaagcataattgtgtcata
TCGACCCGcCGGGGGTTCAATTCCCCCTTGATGCA

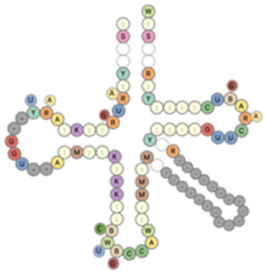

Stigmatella_aurantiaca_DW43-1_Stig_aura_DW4_3_1_tRNA-Gly-GCC-1-1 Myxococcales bacteria (species)

GCGGGAATAGCTCAGCGGTAGAGCATCGCCTTGCCAAGGCGAGGGtCGAGGGTTCA
AATCCCTTTTCCCGCTCCA

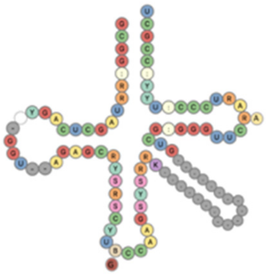

Strongylocentrotus_purpuratus_Spurp5_tRNA-Gly-GCC-4-1 Echinodermata (urchin)

GCATCGGTGGTTCAGTGGTAGAATTCTCGCCTGCCACGCGGGGGaCCCGGGTTCAA
TTCCCGGCCGATGCA

Sulfobacillus_acidophilus_DSM_10332_Sulf_acid_DSM_10332_tRNA-Gly-GCC-1-1 Bacillota

GCGGAAGTAGCTCAGTGGTAGAGCATCGCCTTGCCAAGGCGAGGGtCGCGGGTTCA
AATCCCGTCTTCCGCTCCA

Sulfolobus_islandicus_HVE104_Sulf_isla_HVE10_4_tRNA-Gly-GCC-1-1 Proteoarchaeota (species)

GCGGCCG**T**AGTCTAGCC**TGG**AttAGGACGC**CTGCCTGCCA**CGCAGGAGGtCCCGGG**T**
TCAAATCCCGGCGGCCGCA

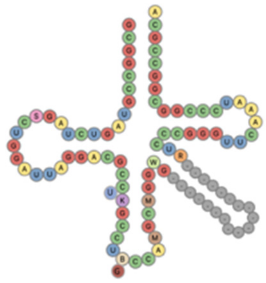

Sus_scrofa_Sscro11_tRNA-Gly-GCC-5-1 Mammals (pig) (species)

GCATGGG**TG**GTTCAGG**GGTA**GAATTCTCAC**CTGCCA**CGTGGGAGGCCCGGG**TTCAA**
TTCCCGGCCTGTGCG

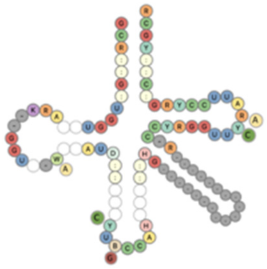

Syntrophobacter_fumaroxidans_MPOB_Synt_fuma_MPOB_tRNA-Gly-GCC-1-1 Thermodesulfobacteriota bacteria (species)

GCGGGA**AT**AACTC**A**GC**GGTA**GAGTGCAACCT**TGCCA**AGGTTGAAGtCGCGGG**TTCAA**
ATCCCGTTTCCCGCTCCA

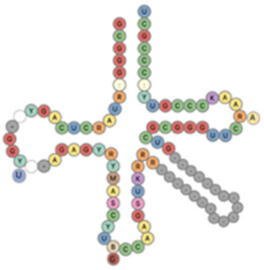

Taeniopygia_guttata_Tgutt2_tRNA-Gly-GCC-10-1 Aves (species)

GCCCTGG**TG**GCTCCG**TGGTA**GAATTCTGC**CTGCCA**CGGCGGCAgCCTGGG**TTCAA**T
TCCCGGCAGAGGCA

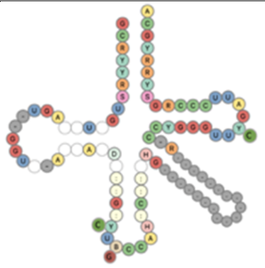

Tarsius_syrichta_Tsyri2_tRNA-Gly-GCC-15-1 mammals (tarsier)

ACATGGGTAGTTCAGTGGTAGAATTCTCGCCTGCCACACAGGAGGCCCGGATTCAATTACTAACCCATGCAT

Tepidanaerobacter_acetatoxydans_Re1_Tepi_acet_Re1_tRNA-Gly-GCC-1-1 Bacillota (species)

GCGGAAGTGGCTCAGTGGTAGAGCATCGCCTTGCCAAGGCGAGGGtCGCGGGTTCAAATCCCGTCTTCCGCTCCA

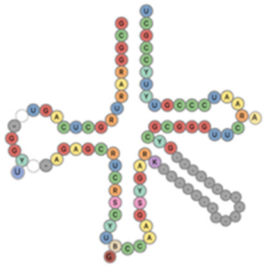

Terribacillus_aidingensis_MP602_Terr_aidi_MP602_tRNA-Gly-GCC-1-1 Bacillota (species)

GCGGAAGTAGTTCAGTGGTAGAACACCACCTTGCCAAGGTGGGGGtCGCGAGTTCAAATCTCGTCTTCCGCTCCA

Terriglobus_roseus_DSM_18391_Terr_rose_DSM_18391_tRNA-Gly-GCC-1-1 Acidobacteriota bacteria (species)

GCGGGAGTAGCTCAGTGGTAGAGTGCTTCCTTGCCAAGGAAGATGtCGCGGGTTCAAATCCCGTCTCCCGCTCCA

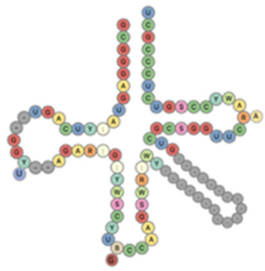

Thaumarchaeota_archaeon_SAT1_Thau_arch_SAT1_tRNA-Gly-GCC-1-1 Thaumarchaeota archaea

GCGGCTGTAGTATAGCCTGGCcAGTACGCGGGATTGCCAATTCTGTGACCCGGGTTCAAATCCCGGCAGCCGCA

Thermacetogenium_phaeum_DSM_12270_Ther_phae_DSM_12270_tRNA-Gly-GCC-1-1 Acidobacteriota bacteria (species)

GCGGAAG**T**AGCTCAGC**GGTA**GAGCATCGCCT**TGCCA**AGGCGAGGGcCGCGGG**TTCAA**ATCCCGTCTTCCGCTCCA

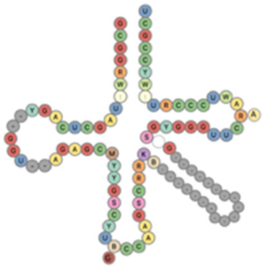

Thermanaerovibrio_acidaminovorans_DSM_6589_Ther_acid_DSM_6589_tRNA-Gly-GCC-1-1 Synergistota bacteria

GCGGAAG**T**AGCTCAGG**GGTA**GAGCACAACCT**TGCCA**AGGTTGGGGtCGCGGG**TTCAA**ATCCCGTCTTCCGCTCCA

Thermococcus_barophilus_MP_Ther_baro_MP_tRNA-Gly-GCC-1-1 Euryarchaeota (species)

GCGGTGG**T**AGTCTAGCC**TGG**CctAGGACAGCGGC**CTGCCA**CGCCGCGGCCCGGG**TTCAA**ATCCCGGCCACCGCACCA

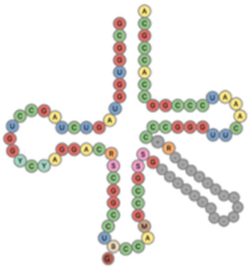

Thermodesulfatator_indicus_DSM_15286_Ther_indi_DSM_15286_tRNA-Gly-GCC-1-1 Thermodesulfobacteriaceae bacteria (species)

GCGGGAG**T**AGCTCAGT**TGGTA**GAGCGCCACCT**TGCCA**AGGTGGATGtCGCGGG**TTCAAG**TCCCGTCTCCCGCTCCA

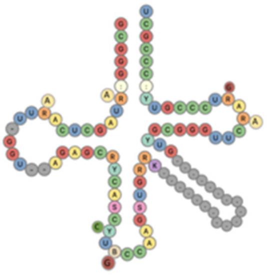

Thermodesulfovibrio_yellowstonii_DSM_11347_Ther_yell_DSM_11347_tRNA-Gly-GCC-1-1 Methanococcaceae archaea

GCGGGTG**T**AGCTCAGC**TGGTA**GAGCACAACCT**TGCCA**AGGTTGGGGtCGCGGG**TTCAA**ATCCCGTCGCCCGCT

Thermofilum_carboxyditrophus_1505_Ther_carb_1505_tRNA-Gly-GCC-1-1 Thermofilaceae archaea

GCGGCCG**T**AGTCTAGTC**TGGTA**GGATGGCGGC**CTGCCA**CGCCGCAGAaCCCGGG**TT**
CAAATCCCGGCGGCCGCACCA

Thermogladius_cellulolyticus_1633_Ther_cell_1633_tRNA-Gly-GCC-1-1 Crenarchaeota

GCGGCGG**T**CGTCTAGCC**TGG**ActAGGACGCCGGC**CTGCCA**CGCCGGAAAtCCCGGG**T**
TCAAATCCCGGCCGCCGCA

Thermoplasma_volcanium_GSS1_Ther_volc_GSS1_tRNA-Gly-GCC-1-1 Thermoplasmataceae archaea (species)

GCGGGTG**TG**GTGTAGCC**TGG**CAACACGCGAGCT**TGCCA**AGCTCGTGcCTCGGG**TTC**
AAATCCCGACATCCGCA

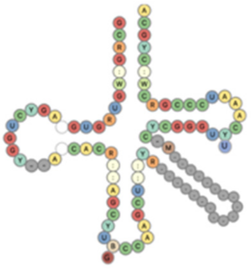

Thermoproteus_neutrophilus_V24Sta_Pyro_neut_V24Sta_tRNA-Gly-GCC-1-1 Thermoproteaceae archaea

GCGGCGG**T**AGTCTAGCC**TGGT**TTGGACGACGGGCGGTCCCGTCGCCCCGGAGTAG
GATGGCGGC**CTGCCA**AGCCGTTGATCCCGGG**TTCAA**ATCCCGGCCGCCGCACCA

Thermosediminibacter_oceani_DSM_16646_Ther_ocea_DSM_16646_tRNA-Gly-GCC-1-1 Bacillota (species)

GCGGAAG**TG**GCTCAGG**GGTA**GAGCATCGCCT**TGCCA**AGGCGAGGGtCGCGGG**TTCA**
AATCCCGTCTTCCGCTCCA

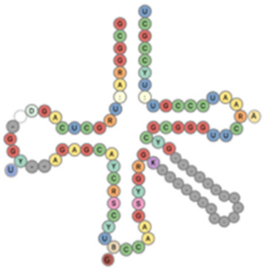

Thermosipho_africanus_TCF52B_Ther_afri_TCF52B_tRNA-Gly-GCC-1-1 Thermotoga bacteria

GCGGGTG**T**AGCTC**A**G**TGGTA**GAGCGCCTGCT**TGCCA**AGCAGGAGGtCGCGGG**TTCA**
AATCCCGTCGCCCGCTCCA

Thermosphaera_aggregans_DSM_11486_Ther_aggr_DSM_11486_tRNA-Gly-GCC-1-1 Proteoarchaeota archaea

GCGGCGG**T**CGTCTAGCC**TGG**ActAGGACGCCGGC**CTGCCA**CGCCGGAAAtCCCGGG**T**
TCAAATCCCGGCCGCCGCA

Thermovibrio_ammonificans_HB-1_Ther_ammo_HB_1_tRNA-Gly-GCC-1-1 Desulfurobacteriaceae bacteria (species)

GCGGGCGTAGCTCAGTTGGTAGAGCGCAACCTTGCCAAGGTTGAGGtCGCGGGTTCAAGTCCCGTCGCCCGCTCCA

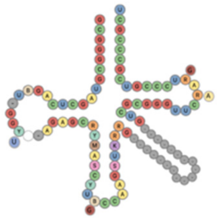

Thermus_oshimai_JL-2_Ther_oshi_JL_2_tRNA-Gly-GCC-1-2 Deinococcota bacteria (species)

GCGGGAGTAGCTCAGTTGGTAGAGCACGACCTTGCCAAGGTCGGGGtCGCGGGTTCAAGTCCCGTCTCCCGCTCCA

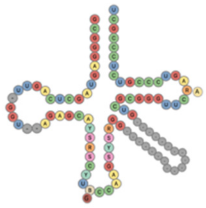

Thermus_scotoductus_SA-01_Ther_scot_SA_01_tRNA-Gly-GCC-1-2 Deinococcota bacteria (species)

GCGGGAGTAGCTCAGTTGGTAGAGCACGACCTTGCCAAGGTCGGGGtCGCGGGTTCAAGTCCCGTCTCCCGCTCCA

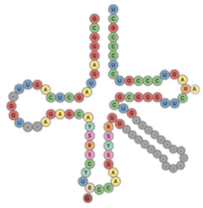

Thuidium_tamariscinum_Ttama2_tRNA-Gly-GCC-2-1 Bryophyta

GCGGAAATAGCTTAATGGTAGAGTATAGCCTTGCCAAGGCTGAGGtTGAGGGTTCAAGTCCCTTTTTCCGCT

Treponema_denticola_ATCC_35405_Trep_dent_ATCC_35405_tRNA-Gly-GCC-1-1 Spirochaetota (species)

GCGGGAATAGCTCAGTGGTAGAGCGCCACCTTGCCAAGGTGGATGtCGCGAGTTCAATCCTCGTTTCCCGCT

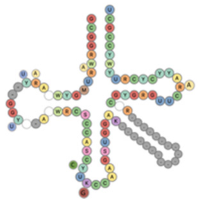

Trichechus_manatus_latirostris_Tmana1_tRNA-Gly-GCC-13456-1 Mammals (manatee) (species)

ACCCTGGTGGCTCAGTGGTtAAAGCGCTTGGCTGCCAACCAAAAGGtCATTGGTTCAAACCCATTagctgctCTGTGGGA

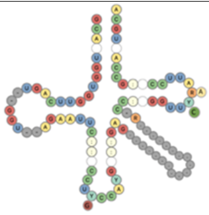

Triticum_aestivum_Taest1_tRNA-Gly-GCC-62-1 Plant (wheat)

GCACCAG**TG**GTCTAG**TGGTA**GAATAGTACC**CTGCCA**TGGTACAGaCCTGGG**TTCAA**T
TCCTGGCTGGTGGA

Turneriella_parva_DSM_21527_Turn_parv_DSM_21527_tRNA-Gly-GCC-1-1 Spirochaetota bacteria (species)

GCGGGA**AT**AGCTC**A**G**TGGTA**GAGCACCTCCT**TGCCA**AGGAGGGGGtCGTGGG**TTCA**
AGTCCCATTTCCCGCT

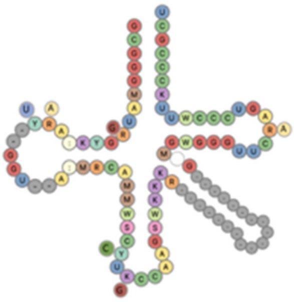

Tursiops_truncatus_Ttrun2_tRNA-Gly-GCC-166-1 Mammals (dolphin) (species)

GCATTGG**TG**ATTC**A**G**TGGTA**GAATTCTTGC**CTGCCA**CGTGGGAGGCCAGGG**TTCAA**T
TCCCAGCCAATGCA

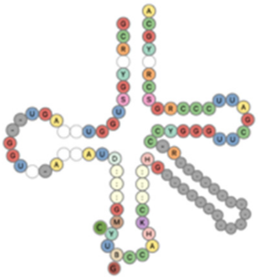

Ustilago_maydis_521_Usti_mayd_521_tRNA-Gly-GCC-2-1 Fungus (species)

GCATTGG**T**AGTGT**A**G**TGGTA**TCACGGGACGT**TGCCA**gcccgcctta**TTCAAG**gcttaaccacga
atTCGTC

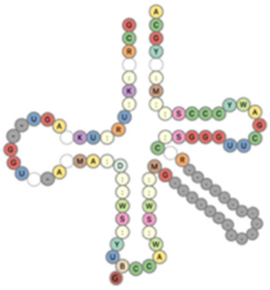

Vicugna_pacos_Vpaco2_tRNA-Gly-GCC-4-1 mammals (alpaca) (species)

GGAGGg**T**ATAGCTC**A**G**TGGTA**GAGTGCATGC**CTGCCA**TGCACAAGGtCCTGGG**TTCA**
ATCCCCAGTACCTCCA

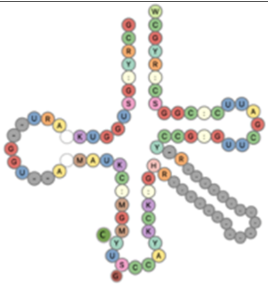

Vitis_vinifera_Vvini_tRNA-Gly-GCC-2-1 Plant (grape) (species)

GCGGAA**AT**AGCTT**AATGGTA**GAGCATAGCCT**TGCCA**AGGCTGAGGtTGAGGG**TTCAAG**TCCCTCCTTCCGCT

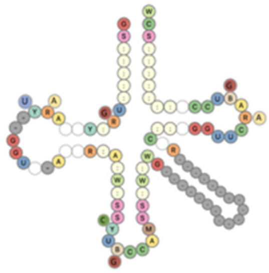

Volvariella_volvacea_V23_Volv_volv_V23_tRNA-Gly-GCC-1-2 Fungus (species)

GCATTA**ATG**GGGTAG**TGGTA**ACCTGGGTCGT**TGCCA**TTCGAttcc**TTCAAG**cTCGACCCGcCGG

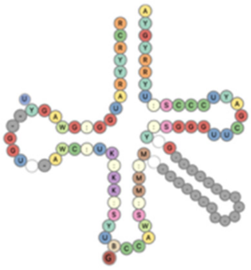

Waddlia_chondrophila_WSU_86-1044_Wadd_chon_WSU_86_1044_tRNA-Gly-GCC-1-1 Waddliaceae bacteria (species)

GCGGGTG**T**AGCTC**A**GC**GGTA**GAGCATCACGT**TGCCA**ACGTGAGGGtCGTGAG**TTCAA**ATCTCATCACCCGCT

Xenopus_tropicalis_Xtrop9_tRNA-Gly-GCC-4-1 Frog (species)

GCATTGG**TG**GTTC**A**G**TGGTA**GAATTCTCGC**CTGCCA**CGCGGGAGGCCCGGG**TTCAA**TTCCCGGCCAATGCA

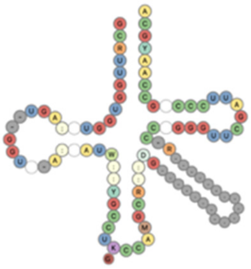

Candidate_division_SR1_bacterium_RAAC1_SR1_tRNA-Gly-GCC-1-1 Bacteria

GCAGGC**AT**AGCTCA**A**T**TGG**CtAGAGCGCTTCCT**TGCCA**AGGAAGAGGtTGCGGG**TTCAA**ATCCCGTTGCTTGCTCCA

Uncultured_Termite_group_1_bacterium_phylotype_Rs-D17_tRNA-Gly-GCC-1-1
Cellulolytic bacteria (species)

GCGGGTG**T**AGTTC**A**G**TGGTA**GAACGTCTCGT**TGCCA**ACGAGAAGGtCGTGGG**TTCAA**
GTCCCATCGCCCGCT

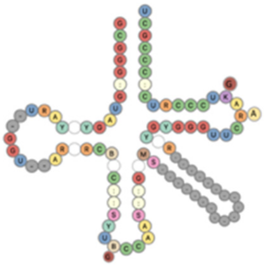

Above list of 246 species is extracted from GtRNAdB (http://lowelab.ucsc.edu/GtRNAdb/), which contains 18,047 tRNA-Gly^{GCC} from 4,857 species: Eukaryota 599 12.3%, Archaea 220 (4.5%) and Bacteria 4,038 (83.1%). The 241 species are the only from the 4,857 species which have both the pentamers TGCCA in the anticodon loop and TTCAA in the Tψ-loop of their tRNA-Gly^{GCC} cloverleaf structure. The high proportion of Archaea (54/246 = 22%) is due here to the primitive character of the AL-decamer *TGCCATTCAA*. The remaining part of AL, *GATGAATGGTAC*, occurs in articulation and D-loop of the cloverleaf.

Table S3. List of nucleolin AL-proximity for 22 species.

22 Species with Nucleolin mRNA	AL-pentamer proximity
Solanum lycopersicum nucleolin (NCL) LOC101260453, mRNA NCBI Sequence: XM_010326160.3	18.1σ
Hydrotalea sandarakina nucleolin (NCL) str. DSM 23241 LX80DRAFT, GenBank: QKZV01000007.1	16.8σ
Gallus gallus nucleolin (NCL), transcript variant X1, mRNA NCBI Sequence: XM_046898333.1	13.2σ
Bauhinia variegata nucleolin (NCL) isolate BV-YZ2020 chromosome 1, GenBank: JAKRYI020000001.1	13σ
Homo sapiens nucleolin (NCL), mRNA NCBI Sequence: NM_005381.3	11.8σ
Lactobacillus lindneri nucleolin (NCL) DSM 20690 = JCM 11027, GenBank: FUXS01000002.1	11.7σ
Hydrobacter penzbergensis nucleolin (NCL) strain DSM 25353, GenBank: FNNO01000007.1	11.5σ
Ornithorhynchus anatinus nucleolin (NCL), mRNA NCBI Sequence: XM_029064618.2	11σ
Acanthochromis polyacanthus nucleolin (NCL) LOC110950732, mRNA NCBI Sequence: XM_022193488.2	10.8σ
Bactrocera dorsalis nucleolin (NCL) Fly_Bdor chr. 5 ASM2337382v1, mRNA NCBI Sequence: NC_064307.1	10.7σ
Monodelphis domestica nucleolin (NCL), variant X2, mRNA NCBI Sequence: XM_056819948.1	9.4σ
Xyrauchen texanus nucleolin (NCL) LOC127639553, mRNA NCBI Sequence: XM_052121621.1	9.3σ
Cannabis sativa nucleolin (NCL) chromosome 1, cs10, mRNA NCBI Sequence: NC_044371.1	9.3σ
Xenopus laevis nucleolin L homeolog (NCLL), mRNA NCBI Sequence: NM_001372137.1	8.8σ
Arabidopsis thaliana nucleolin like 2 (NUC-L2), mRNA NCBI Sequence: NM0_01338347.1	7.5σ
Dicentrarchus labrax nucleolin (NCL) LOC127349869, mRNA NCBI Sequence: XM_051375928.1	6.9σ
Helianthus annuus nucleolin (NCL) cultivar HA300 chromosome 17, GenBank: JANJOV010001181.1	6.9σ
Exophiala nucleolin (NCL) sp. JF 03-4F unplaced genomic scaffold EDD36 scaffold_3, GenBank: MU404352.1	6.8σ
Raphanus sativus nucleolin-like (NCL) cultivar WK10039 chromosome 4, GenBank: JRUI03000004.1	6.5σ
Cyprinus carpio nucleolin-like (NCL) LOC109082092, mRNA NCBI Sequence: XM_042712222.1	6.25σ
Carex littledalei nucleolin (NCL) isolate C.B.Clarke chromosome 3, GenBank: SWLB01000003.1	5.6σ
Saccharomyces cerevisiae nucleolin (NCL) S288C chr. VII, mRNA NCBI Sequence: NC_001139.9	4.6σ

**Table S4.** Clusters obtained by Maxwell© from Archaea genomes [34]	Distance to Cluster barycenter	AL-proximity
Palaeococcus pacificus DY20341	0	63.58
Thermoplasmateles archaeon SM1-50	1000130	25.7
Thaumarchaeota archaeon JGI OTU-3	1000407	2.19
Candidate divison MSBL1 archaeon SCGC-AAA259J03	1000185	10.7
Thermoproteus tenax Kra1	1019727	-8.5
Methanothermus fervidus DSM2088	1012772	91.34
Ignicoccus hospitalis KIN4 I	1015594	6.54
Ferroglobus placidus DSM 10642(2)	1006762	35,3
Candidatus Korarchaeum cryptofilum OPF8	1014796	64.98
Thermococcus kodakarensisKOD1	1003734	34.52
Pyrococcus furiosus DSM3638	996602	78.2
Methanopyrus kandleri AV19	999896	-3.1
Halorubrum lacusprofundi ATCC 49239	0	-82
Natrinema pellirubrum DSM 15624	997811	-95,5
Halomicrobium mukohataei DSM 12286	996207	-91,5
Methanofollis liminatans DSM 4140	0	-17,7
Methanoculleus bourgensis MS2	999743	-16,6
Aciduli profundum boonei T469	0	85,5
Aciduli profundum SP Mar 08-339	997107	72,3
Archaeoglobus veneficus SNP6(2)	0	28,5
Archaeoglobus fulgidus	999098	35,2
Sulfolobus acidocaldarius	0	59,8
Sulfolobus tokodaii str.	999336	99.52
Methanocaldococcus jannaschii	0	9,16
Methanocaldococcus jannaschii DSM2661	127503	9.87
Hadesarchaea archaeon DG-33	0	10.49
Hadesarchaea archaeon YNPN2	999702	8.82
Candidate divison MSBL1 archaeon SCGC-AAA259A05	0	2.56
Candidate divison MSBL1 archaeon SCGC-AAA259E19	997758	4.85
Dehydroabietic acid-degrading bacterium	0	-0,4
Leptothrix sp	403141	-2.24
Candidatus Nitrosopelagicus brevis	0	136,8
Candidatus Nitrosopumilus koreensis AR1	991517	149,6
Candidatus Bathyarchaeta archaeon B25	0	2,35
Candidatus Bathyararchaeta archaon B26	975089	1,034
Altiarchaeales archaeon SM1	0	28.64
Candidatus altiarchaeales	293	35.09
Candidatus Methanoplasma termitum	0	40,2
Vulcanisaeta moutnovskia 768-28	0	43.58

Thiobacillus denitrificans copie	0	-6,8
Thorarchaeota archaeon SMTZ1-83	0	17.48
Thermoplasma volcanium GSS1	0	71,28
Thorarchaeota archaeon SMTZ-45	0	32.34
Thermoplasmatales archaeon DG-70-1	0	26.78
candidatus caldiarchaeum subterraneum	0	14,5
Thermoplasmatales archaeon SM1-50	0	25,7
ThermoplasmatalesarchaeonDG-70	0	18.4
Thermoplasmatales archaeon SG8-52-4	0	-0,09
Thermoplasmatales archaeon SG8-52-3	0	39
Thermofilum pendens Hrk5	0	-42.72
Thermoplasmatales archaeon SCGC AB-539	0	16
Thermoplasmatales archaeon E-plasma	0	86,2
Thermoplasmatales archaeon I-plasma	0	52,38
Thermoplasmatales archaeon A-plasma	0	22,07
Pyrolobus fumarii	0	-20,7
Candidatus Methanomethylophilus alvus Mx1201	0	23,3
Thaumarchaeota archaeon SCGC AAA007-O23	0	29,46
Thaumarchaeota archaeon JGI OTU-1	0	10,2
Thaumarchaeota archaeon SCGCAB-179	0	7.79
Nitrospina gracilis	0	18,2
Sinorhizobium	0	-2.19
Methylocaldum szegediense	0	-6,04
Methanosarcina mazei Go1	0	87
Methanotorris igneus kol5	0	112.25
Nitrospina sp	0	10,87
Methanoregula boonei 6A8	0	1,65
Candidatus Methanoperedens nitroreducens	0	20
Methylococcus capsulatus str. Texas	0	-15,7
Methanothermococcus thermolithotrophicus DSM2095	0	101.85
Methermicoccus shengliensis DSM 18856	0	0,35
Methanocorpusculum labreanum Z	0	43,36
Methanomethylicus mesodigestum V2	0	7.79
Methanomethylicus oleusabulum V2	0	3.67
Methanococcoides burtonii DSM 6242	0	99,44
Methanocella arvoryzae MRE50	0	-2,2
Methanocella paludicola SANAE	0	-2 ,2
Methanocella conradii HZ254	0	5,95
Caldisphaera lagunensis DSM 15908	0	90.66
Methanococcus vannielii SB	0	119.36

Methanobrevibacter smithii ATCC35061	0	119.46
Methanobacterium formicicum	0	79.77
Metallosphaera sedula DSM5348	0	57.06
Haloferax mediterranei ATCC 33500	0	-58
Lokiarchaeum SP.GC1475	0	33.04
Hyperthermus butylicus DSM5456	0	-22.82
Hyphomicrobium	0	-15.23
Ferroplasma acidarmanus Fer1	0	79,7
Geoglobus ahangari	0	21.81
Altiarchaealesarchaeon IMC4	0	37.8
Canditatus Nitrosocosmius MY3	0	165,7
HadesarchaeaarchaeonYNP45	0	4.89
Dunaliella salina	0	113
Cenarchaeum symbiosum A	0	-4,4
Candidatus Odinarchaeota archaeon LCB_4	0	61.03
Euryarchaeota archaeon SCGCAAA252-115	0	19.37
Diplonema papillatum	0	-8,3
Candidatus Nitrosotenuis cloacae	0	69,2
Desulfurococcus mobilis	0	1,58
Crenarchaeota JGI-OTU-1	0	34.63
Candidatus Bathyarchaeota archaeon B24	0	0,33
Crenarchaeota groupe-15 archeon	0	3,625
Candidatus Lokiarchaeota archaeon CR_4	0	16.81
Candidatus Heimdallarchaeota archaeon AB_125	0	19.11
Candidatus Heimdallarchaeota archaeon LC_3	0	39.4
Candidatus Syntrophoarchaeum butanivorans	0	43,05
Candidatus Thalassoarchaea betae	0	6,62
Candidatus Heimdallarchaeota archaeon LC_2	0	40.15
Candidatus alkanophagales	0	-1.02
Candidatus Syntrophoarchaeum CALDARIS	0	24,22
Candidatus Proteinoplasmatales archaeon SG8-5	0	0,09
Candidate division MSBL1 archaeon SCGC-AAA259D14	0	8.3
Aquifex pyrophilus	0	0,032
Thermococcus sibiricus MM739	0	-3.59

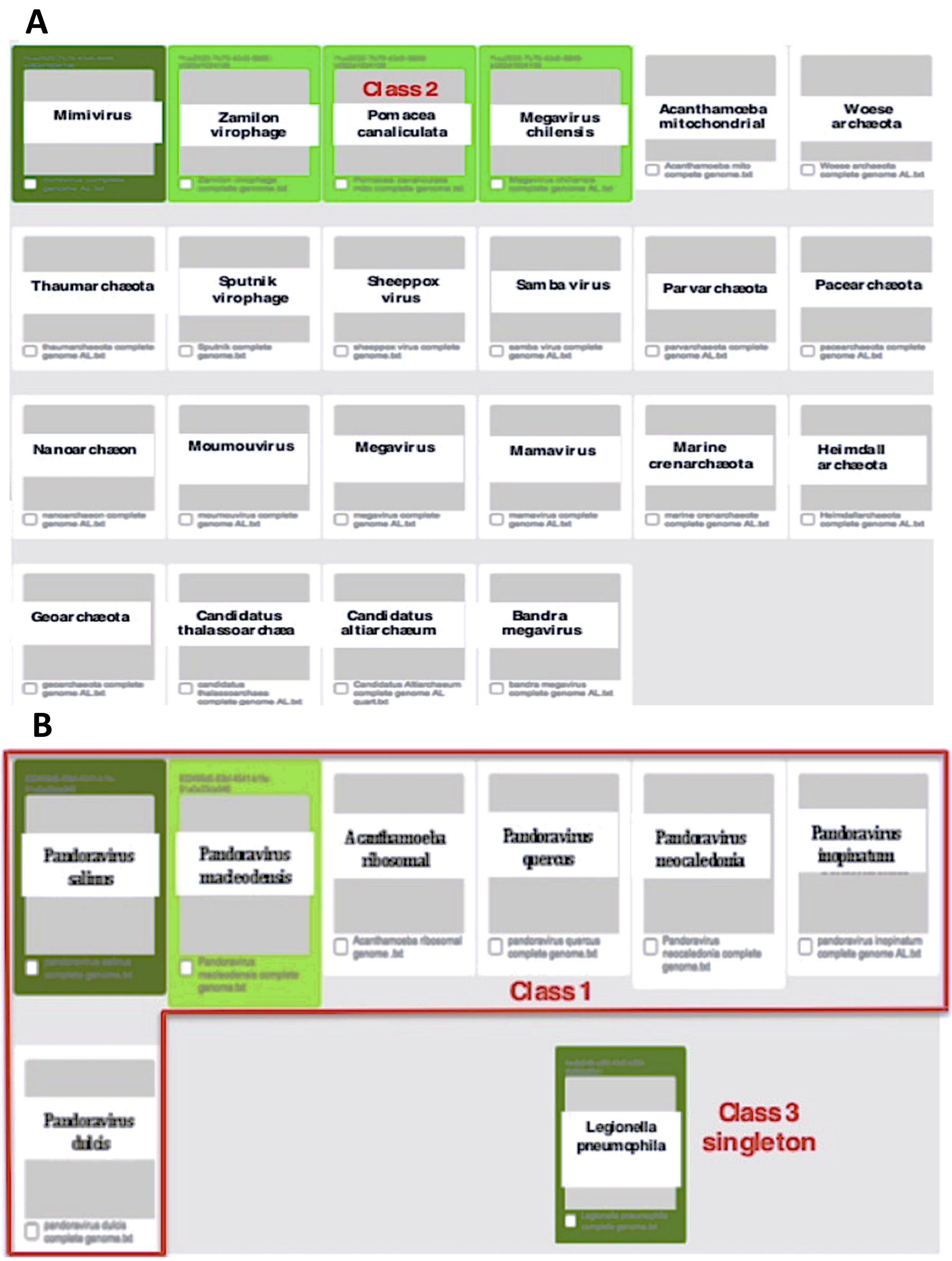

Figure S2. A) Class 2 from the classification by Maxwell of the Giant viruses, their virophages and infectious targets showing together Mimivirus, its virophage Zamilon and its target Acanthamoeba. B) Class 1 of the same classification bringing together genomes of the Pandora family plus its target Acanthamoeba castellanii. The class 3 contains a Bacterium Legionella pneumophila, for which Acanthamoebae serve as vehicles and hosts (see Gomes TS, Gjiknuri J, Magnet A, Vaccaro L, Ollero D, Izquierdo F, Fenoy S, Hurtado C, Del Águila C. The Influence of Acanthamoeba-Legionella Interaction in the Virulence of Two Different Legionella Species. Front Microbiol. 2018, 9:2962). A change of distance threshold causes the entry of the singleton into the class 1, proving their co-evolution.

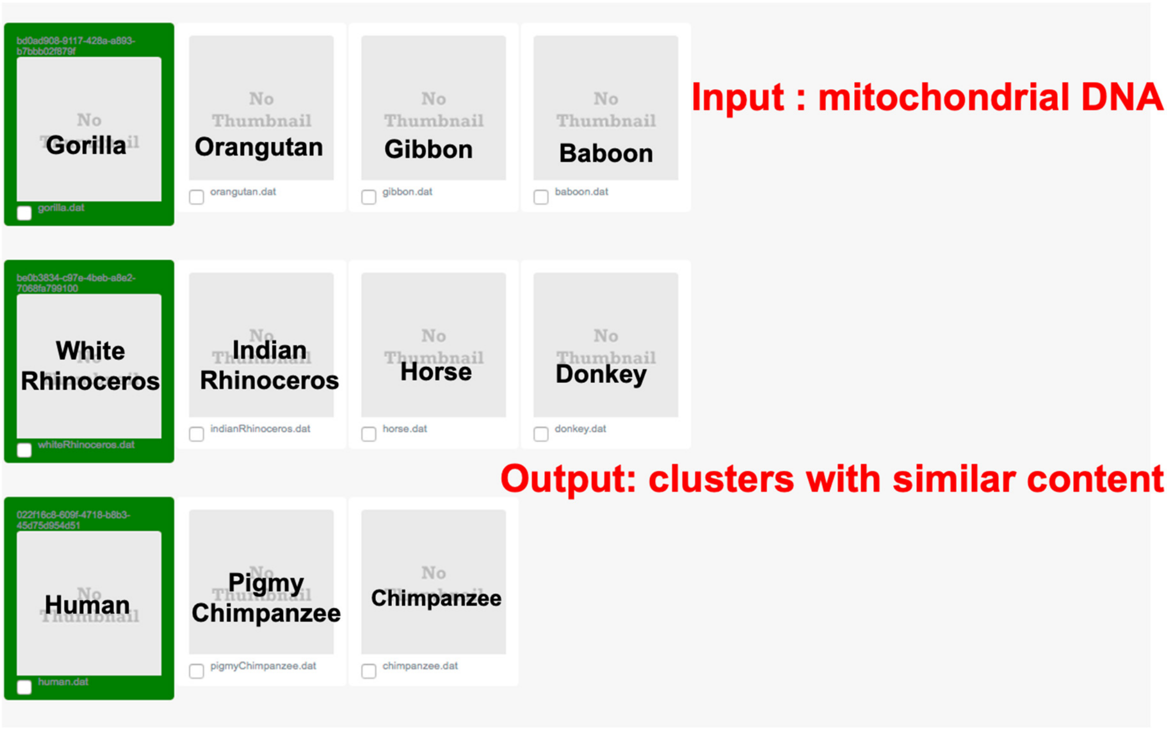

Figure S3. The classification by Maxwell of mammals from their mitochondrial DNA is coherent with the classical knowledge about their evolution.
